# Peptide Sequence Programmed Piezoelectric Response by Supramolecular Self‐Assembly

**DOI:** 10.1002/advs.202515237

**Published:** 2025-12-15

**Authors:** Xuejiao Yang, Shuaijie Liu, Honglei Lu, Yuehui Wang, Hongyue Zhang, Wei Ji, Huaimin Wang

**Affiliations:** ^1^ Westlake Laboratory of Life Sciences and Biomedicine 18 Shilongshan Road Hangzhou Zhejiang 310024 China; ^2^ Department of Chemistry School of Science Westlake University Institute of Natural Sciences Westlake Institute for Advanced Study No. 600 Dunyu Road Hangzhou Zhejiang 310024 China; ^3^ Key Laboratory of Biorheological Science and Technology Ministry of Education College of Bioengineering Chongqing University Chongqing 400044 China

**Keywords:** crystals, peptide, piezoelectricity, power generation, self‐assembly

## Abstract

Piezoelectricity has attracted significant attention for its ability to convert mechanical energy into electricity, especially when biological molecules are incorporated into materials. However, natural biomaterials are limited by low piezoelectric coefficients, necessitating synthetic alternatives. Short peptides offer advantages such as ease of synthesis, biocompatibility, and tunability, making them promising candidates. In this study, hydrophobic tripeptides are used to fabricate stable piezoelectric crystals. When phenylalanine occupies the central position, the tripeptides self‐assembled into uniform, thermally stable crystals. Piezoresponse force microscopy (PFM) reveals a high effective piezoelectric coefficient of 24.0 pC N^−1^, attributed to strong asymmetric molecular packing, confirmed by microcrystal electron diffraction (MicroED) and density functional theory (DFT) analysis. Nanogenerators based on these crystals achieve an open‐circuit voltage of 2.57 V under 50 N, the highest among short peptides in aqueous environments. These findings advance peptide crystal engineering and highlight their potential for high‐performance piezoelectric devices.

## Graphical abstract

In this study, hydrophobic tripeptides are explored as building blocks for stable piezoelectric crystals. Tripeptides with phenylalanine at the central position self‐assembled into uniform, thermally stable crystals. Piezoresponse force microscopy reveals a high effective piezoelectric coefficient of 24.0 pC N^−1^, attributed to asymmetric molecular packing confirmed by MicroED and DFT analysis. Using these crystals in nanogenerators, an open‐circuit voltage of 2.57 V is achieved under 50 N force‐the highest reported for short peptides in aqueous environments. This work advances peptide crystal engineering and offers a promising route for high‐performance piezoelectric devices.

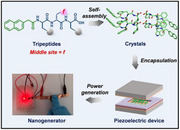

## 1 Introduction

Piezoelectric materials possess the remarkable ability to convert mechanical force into electric energy and conversely to vibrate under the influence of an electric field. The piezoelectric response exhibits a linear relationship with the applied electric field.^[^ ^1^, ^2^, ^3^, ^4^, ^5^ ^]^ The development of piezoelectric materials has predominantly focused on inorganic compounds and organic polymers, such as barium titanate (BaTiO_3_),^[^ ^6^ ^]^ lead zirconate titanate (PZT),^[^ ^7^ ^]^ and polyvinylidene fluoride (PVDF).^[^ ^8^ ^]^ These materials have been extensively utilized in the fabrication of piezoelectric transducers, actuators, and intelligent robotics.^[^ ^9^, ^10^, ^11^, ^12^ ^]^ Unfortunately, intricate fabrication processes and the use of toxic materials severely hinder their application in the health and life sciences.^[^ ^13^, ^14^ ^]^ In this context, biological materials emerge as highly promising candidates due to their excellent sensitivity, flexibility, and durability, offering a viable strategy for the development of biocompatible energy harvesters. Notably, biological materials such as collagen, bone, hair, elastin, viruses, and DNA have been demonstrated to possess piezoelectric properties.^[^ ^15^, ^16^, ^17^, ^18^ ^]^ However, the measured piezoelectric response of these biological materials typically falls within the range from 0.1 to 10 pC N^−1^, significantly lower than that of commercially available inorganic piezoelectric materials, limiting the potential for fabricating piezoelectric devices from biological materials. Therefore, there is a pressing need to explore novel approaches to enhance the piezoelectric performance of biological materials, unlocking their full potential and widening their scope of applications.

Biomimetic self‐assembly represents an effective strategy for fabricating novel and multifunctional materials.^[^ ^19^, ^20^, ^21^, ^22^, ^23^, ^24^, ^25^, ^26^, ^27^, ^28^, ^29^, ^30^, ^31^, ^32^, ^33^, ^34^, ^35^, ^36^, ^37^, ^38^, ^39^, ^40^ ^]^ By harnessing a minimalistic approach, amino acids and short peptides serve as versatile building blocks endowed with inherent intermolecular hydrogen bonding, *π–π* stacking, and hydrophobic interactions.^[^ ^41^, ^42^, ^43^, ^44^, ^45^, ^46^ ^]^ Moreover, the inherent biocompatibility and versatility of these building blocks impart tailored functionality to the self‐assembled materials, thereby broadening their potential applications. Piezoelectricity has a well‐established third‐rank tensor property, with the piezoelectric response dependent on crystallographic directions.^[^ ^18^ ^]^ Consequently, the formation of crystalline structures is essential for the development of piezoelectric materials. Through the deliberate design and controlled self‐assembly of these building blocks, it is possible to create precisely engineered crystalline structures, enabling the realization of efficient piezoelectric properties.

Amino acids and short peptides have been identified as exhibiting piezoelectric properties.^[^ ^47^, ^48^ ^]^ Notably, γ‐glycine has undergone rigorous testing using PFM, revealing longitudinal piezoelectric responses of 9.9 pC N^−1^.^[^ ^18^, ^49^ ^]^ Additionally, L‐tyrosine has been shown to self‐assemble into crystals with a piezoelectric response of 9.7 pC N^−1^.^[^ ^50^ ^]^ Diphenylalanine (FF), a core self‐assembly motif found in the Alzheimer's β‐amyloid polypeptide, is a potent building block capable of directing self‐assembly behaviors. Upon forming hexagonal crystals, FF exhibits a piezoelectric coefficient of 9.9 pC N^−1^,^[^ ^51^ ^]^ which can be enhanced to an impressive value of 17.9 pC N^−1^ by applying an electric field during the crystal growth process.^[^ ^52^ ^]^ These findings highlight the potential of amino acids and short peptides as versatile and tunable building block for fabricating high‐performance piezoelectric materials.

**Figure 1:**
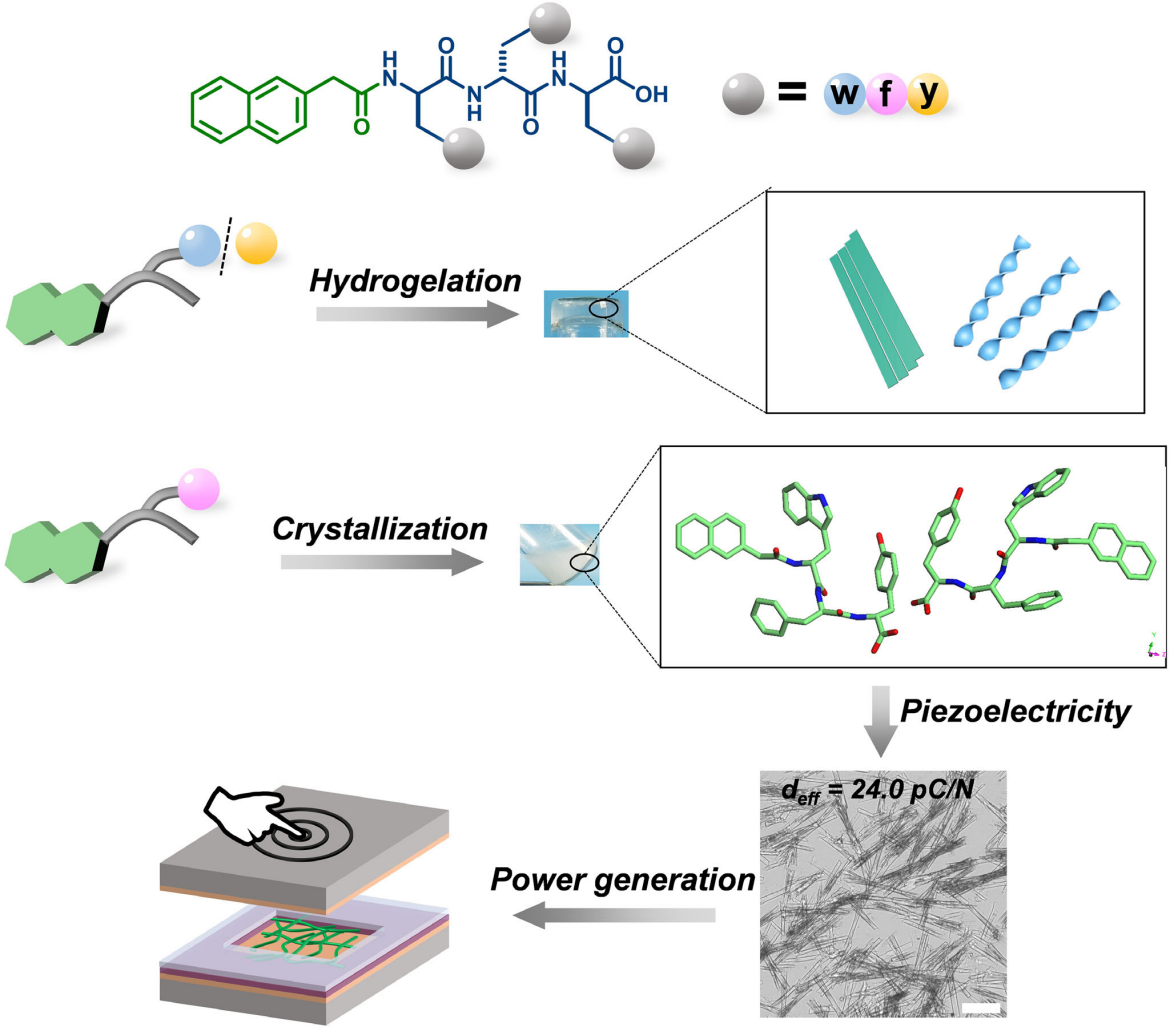
Schematic illustration of the self‐assembly property of **Nap‐X_1_w(y)X_2_** and **Nap‐X_1_fX_2_**, and the piezoelectric application of self‐assembled crystals. The blue, pink, and yellow ball represents w, f, and y, respectively. The X_1_ and X_2_ indicate the amino acid. Scale bar is 50 µm.

Despite the demonstrated piezoelectric properties of amino acid and short peptide‐based materials, several critical challenges remain. First, it is uncertain whether crystal formation can be achieved through the rational design of short peptide self‐assembly. Second, methods to enhance the piezoelectricity of peptide‐based materials require further exploration. Consequently, our research aims to address these challenges by developing self‐assembled peptide crystals with exceptional piezoelectric characteristics. We present here that simple modifications in the peptide sequence can lead to significant alterations in the resulting supramolecular self‐assembly morphologies and microstructures (**Figure** **1**). This achievement is substantiated by the optical images, scanning electron microscope (SEM), transmission electron microscope (TEM), and atomic force microscope (AFM).

Subsequently, we employed PFM to investigate the piezoelectric responses of the self‐assembled peptide crystals. Comparative analysis with previously reported peptide‐based materials revealed that the **Nap‐wfy** crystals exhibited a notably high piezoelectric constant of 24.0 pC N^−1^. To further validate the stacking modes of these crystals, we conducted MicroED, confirming the highly unsymmetric molecular packing within the crystals. Additionally, piezoelectric coefficients obtained from DFT calculations further support our observations in piezoelectricity. We also successfully fabricated sandwiched nanogenerators utilizing the **Nap‐wfy** crystals, which consistently delivered stable power outputs, with exceptional open‐circuit voltage reaching as high as 2.57 V. Notably, this achievement represents the high value reported among peptide‐based materials prepared in aqueous solvents.^[^ ^3^ ^]^ These remarkable outcomes underscore the efficacy of our rational peptide sequence design approach in obtaining crystals with exceptional piezoelectric properties. Our findings offer invaluable inspiration and potential avenues for the development of piezoelectric materials based on short peptides self‐assembly.

## 2 Results

### 2.1 Growth of Dispersive Crystals through Manipulating Peptide Sequence

Our primary objective is to systematically screen a subset of peptides capable of self‐assembling into supramolecular nanostructures with sequence‐dependent properties. Our previous study demonstrated that N‐terminal modification of the tripeptide with naphthylacetic acid (Nap) leads to distinct self‐assembly behaviors: the L‐configured tripeptide forms isotropic nanostructures, whereas its D‐configured counterpart self‐assembles into anisotropic nanostructures.^[^ ^53^ ^]^ In line with previous studies, our focus has centered on D‐tripeptides due to the formation of well‐aligned anisotropic nanostructures. The peptides incorporate aggregation‐prone aromatic amino acids, including phenylalanine (f), tyrosine (y), and tryptophan (w). Utilizing solid‐phase peptide synthesis (SPPS), we synthesized all six tripeptides and subsequently purified them using high‐performance liquid chromatography (HPLC) equipped with a C18 column (Figures S1–S6, Supporting Information). We then proceeded to investigate the self‐assembly properties of these six peptides. Through the process of annealing, involving temporary heating followed by cooling to room temperature, the six peptides exhibited distinctive appearances, indicative of their diverse self‐assembly behaviors. Importantly, all six peptides demonstrated self‐assembly capabilities. Specifically, when the middle amino acids were y and w, the four peptides, including **Nap‐wyf**, **Nap‐fyw**, **Nap‐fwy**, and **Nap‐ywf,** formed transparent hydrogels (Figure S7, Supporting Information). In contrast, when the middle amino acid was f, the peptides **Nap‐yfw** and **Nap‐wfy** self‐assembled into precipitates composed of needle‐like structures. These findings provide clear evidence of the sequence‐dependent self‐assembly properties of the investigated peptides, agreeing with the previous report.^[^ ^21^ ^]^ Moreover, control experiments using peptides without the Nap modification demonstrate a complete absence of crystalline structures (Figures S6, S8–S10, Supporting Information), underscoring the essential role of the Nap group in directing molecular packing and enabling crystal formation. This highlights the synergistic interplay between peptide sequence and aromatic functionalization in governing supramolecular organization.

**Figure 2:**
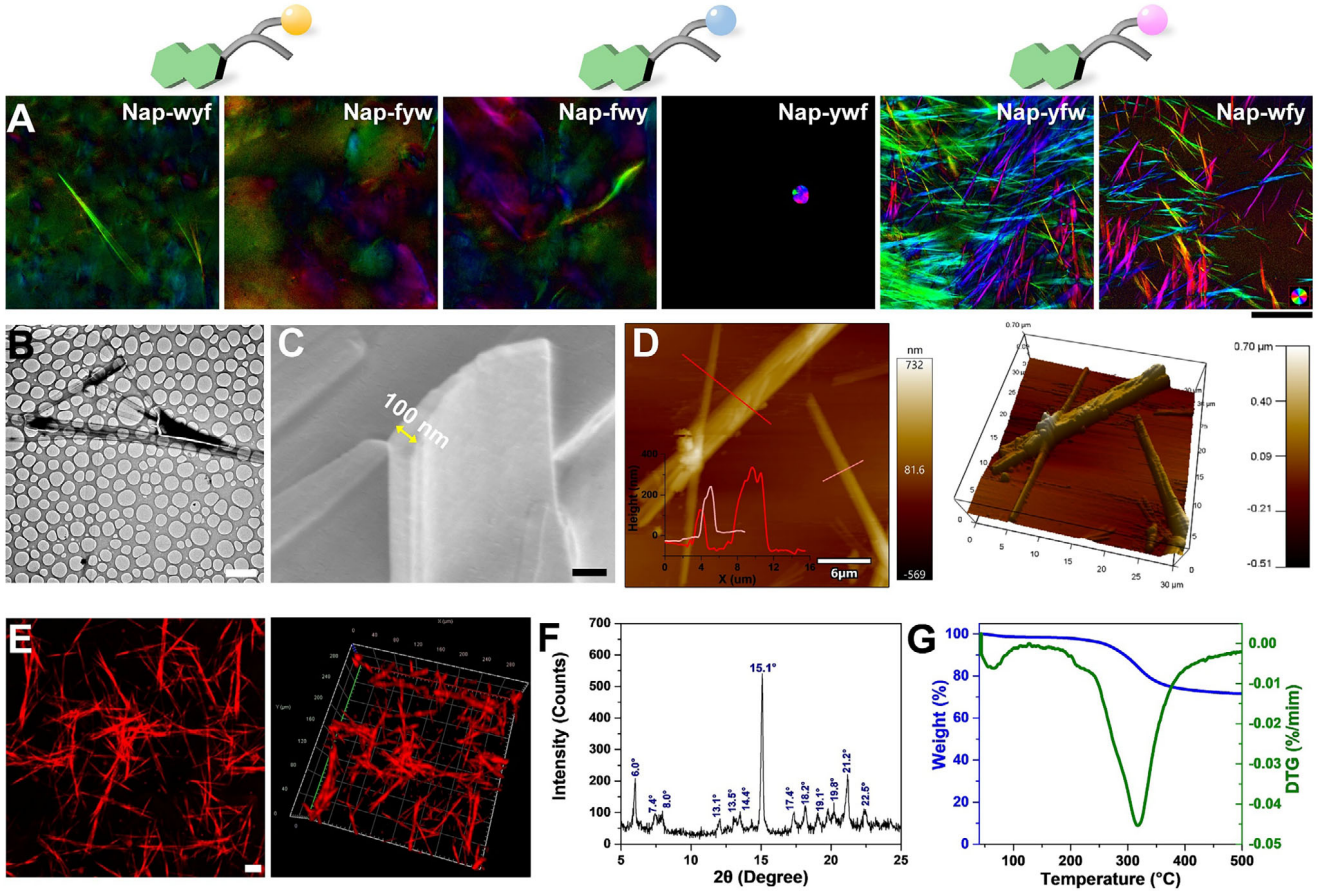
A) Birefringence property of nanostructures self‐assembled by **Nap‐X_1_w(y)X_2_** and **Nap‐X_1_fX_2_**. B) TEM image, C) SEM image, D) AFM image, E) Congo red staining CLSM image, F) PXRD analysis, and G) TGA analysis of **Nap‐wfy** crystals. Scale bar in A), B), C), and E) is 500 µm, 5 µm, 200 nm, and 20 µm, respectively.

The determination of nanoscale morphologies was accomplished using electron microscopes, further revealing a clear dependence of nanostructure morphology on the peptide sequence. Notably, SEM and TEM images showed that **Nap‐wyf** self‐assembled into bundles that consisted of well‐aligned nanofibers exhibiting weak birefringence (**Figure** **2**A; Figures S11A and S12A, Supporting Information). **Nap‐fyw** formed right‐handed nanofibers with an average diameter of 11 nm, displaying no discernible birefringence (Figure 2A; Figures S11B and S12B, Supporting Information). Furthermore, **Nap‐fwy** and **Nap‐ywf** self‐assembled into hydrogels comprised of right‐handed nanofibers, with average diameters of 22 and 13 nm (Figures S11C,D and S12C,D, Supporting Information), respectively, which were confirmed by SEM and TEM images. Remarkably, neither of these two tripeptides exhibited significant birefringence (Figure 2A). However, when the middle amino acid in the tripeptide was f, **Nap‐yfw** and **Nap‐wfy** underwent self‐assembly into crystals, which exhibited pronounced birefringence signals in different directions (Figure 2A–C; Figures S11E,F and S12E,F, Supporting Information). Specifically, the crystals formed by **Nap‐yfw** and **Nap‐wfy** displayed average dimensions of 173 µm in length, 990 nm in width, and 88 µm in length, 780 nm in width, respectively, as confirmed by SEM imaging (Figure S13, Supporting Information). We used AFM to further investigate the height of crystals, and the thickness of crystals was detected as 300 and 100–300 nm for **Nap‐yfw** and **Nap‐wfy** (Figure 2D; Figure S14, Supporting Information), respectively. These comprehensive characterizations provide compelling evidence of the diverse nanoscale morphologies resulting from peptide self‐assembly. The observed variations in fiber alignment, hydrogel formation, and crystal growth highlight the critical role played by specific peptide sequences in dictating the resulting nanostructures.

To gain insights into the packing pattern of tripeptides within the self‐assembled hydrogels and crystals, we first employed circular dichroism (CD) spectra and Fourier transform infrared spectroscopy (FTIR) to investigate the secondary structure of these supramolecular nanostructures. Remarkably, all hydrogels exhibited nearly identical CD spectra, characterized by a negative band at 193–195 nm and a positive band at 210–212 nm; there were signatures diagnostic of a well‐ordered β‐sheet secondary structure (Figure S15A,B, Supporting Information). In contrast, the CD spectra of the crystals formed by **Nap‐yfw** and **Nap‐wfy** displayed a pronounced redshift of the positive band to 220 nm, which can be attributed to a strong *π–π* ^*^ electronic transition arising from the ordered stacking of aromatic moieties.^[^ ^54^ ^]^ This spectral shift suggests that the secondary structure in the crystalline state is composed of a combination of β‐sheet and random coil conformations. Furthermore, distinct and intensified CD signals corresponding to the Nap moiety were observed in the crystalline samples (Figure S15C, Supporting Information), providing direct spectroscopic evidence that the Nap group actively participates in the molecular packing and contributes to the stabilization of the crystalline architecture. These results highlight the critical role of aromatic interactions in directing the supramolecular organization of the peptide crystals. The secondary structure findings were further supported by FTIR analysis. Specifically, in the amide II region, we identified peaks of hydrogels and crystals at 1675–1678 cm^−1^ and 1638–1640 cm^−1^ (Figure S15D–F, Supporting Information), which serve as additional evidence for the β‐sheet secondary structure. To further validate the β‐sheet secondary structure formed inside hydrogels and crystals, we employed Congo red, a well‐known β‐sheet indicator,^[^ ^55^ ^]^ after co‐incubating with the formed hydrogels and crystals, it exhibited strong red fluorescence (Figure 2E; Figure S16, Supporting Information), providing further confirmation of the β‐sheet secondary structure. The consistent CD signals, FTIR peaks, and Congo red fluorescence collectively support the presence of well‐defined β‐sheet motifs, underscoring the robust self‐assembly and structural integrity of the examined peptide nanostructures.

Based on the above analysis on morphology and secondary structure, we then gain further insights into the molecular packing inside crystals and thermostability. We employed powder X‐ray diffraction (PXRD) to analyze the crystal phase. The hydrogels formed by **Nap‐wyf**, **Nap‐fyw**, **Nap‐fwy**, and **Nap‐ywf** exhibited a typical amorphous bulge in their PXRD patterns (Figure S17A,B, Supporting Information), and the diffraction peaks were observed for **Nap‐fyw** and **Nap‐wyf** at 2*θ* values corresponding to a *d*‐spacing of 3.72 and 3.83 Å, respectively. These peaks indicate the presence of *π–π* stacking interactions within the nanostructures. In the case of hydrogels formed by **Nap‐fwy** and **Nap‐ywf**, weak peaks were detected at 2*θ* values of 19.1°, 23.2°, and 23.9°, as well as 19.7°, 23.2°, and 23.9°, respectively. These peaks are associated with *d*‐spacings of 4.64, 3.72, 3.83 Å, and 4.50, 3.72, 3.83 Å, respectively. The presence of these peaks indicates the involvement of hydrogen bonding and *π–π* stacking interactions within the nanostructures formed by **Nap‐fwy** and **Nap‐ywf**. The PXRD diffraction patterns of the hydrogels overall revealed an amorphous and long‐range disordered nature. In contrast, the crystals formed by **Nap‐yfw** and **Nap‐wfy** exhibited a characteristic peak at 2*θ* value of 15.1°, corresponding to a periodic distance of 5.86 Å (Figure 2F; Figure S17C, Supporting Information). This peak suggests a short‐range ordered molecular pattern within the crystalline state, indicating the potential for fabricating crystals‐based piezoelectric materials. These PXRD analyses provide crucial information regarding the molecular arrangements within the investigated hydrogels and crystals. Especially, the crystals exhibit the short‐range ordered patterns, which may benefit for the further applications.

To further assess the thermal stability of the crystals, we conducted thermogravimetric analysis (TGA) to investigate the **Nap‐wfy** crystals. As depicted in Figure 2G and Figure S18 (Supporting Information), the TGA profile revealed that the **Nap‐wfy** crystals exhibit a thermally stable temperature range of 43.0–243.3 °C, while those of **Nap‐yfw** range from 63.0 to 212.9 °C. These results demonstrate that both peptide‐based crystals possess considerable thermal stability, with **Nap‐wfy** displaying a broader stability profile, indicative of superior structural integrity under thermal stress. Combing the above results, it provides compelling evidence for the impact of peptide sequence on the intermolecular interactions, secondary structure, as well as molecular packing inside the self‐assembled nanostructures, which could contribute to the design and synthesis of novel peptide‐based materials with tailored functionalities.

### 2.2 Single‐Crystal Structure of Nap‐wfy Assemblies

**Figure 3:**
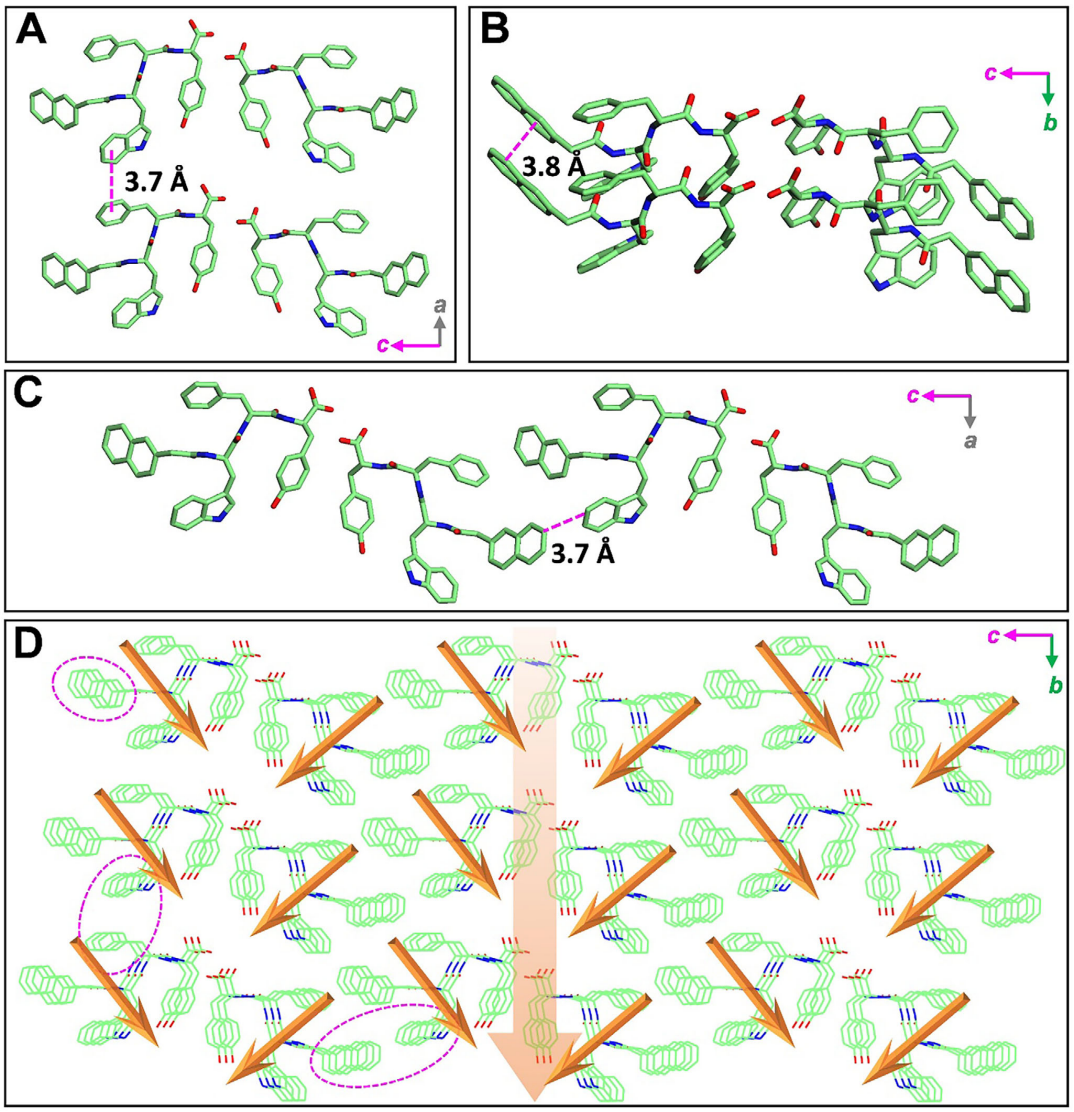
Single crystal structure of triclinic **Nap‐wfy**. The detailed *π–π* stacking interactions between monomers in A) *a* axis, B) *b* axis, and C) *c* axis. D) Molecular packing model of 27 lattices. The pink ellipse curve indicates intermolecular *π–π* stacking interaction between **Nap‐wfy** monomers. The orange arrow indicates the arrangement model of dipole moments within the lattice.

To gain deeper insights into the molecular packing arrangements within the **Nap‐wfy** crystals, we employed microED to resolve their crystal structures. The comprehensive crystallographic data collection and refinement parameters are outlined in Tables S1–S3 (Supporting Information), providing a rigorous foundation for our analysis. The analysis revealed that the crystals formed by **Nap‐wfy** crystallize in a triclinic space group with unit cell parameters of *a* = 4.868 Å, *b* = 13.737 Å, *c* = 29.33 Å, *α* = 99.36°, *β* = 90.24°, *γ* = 90.20°, and a unit cell volume of *V* = 1935 Å^3^. The asymmetric unit contains two independent molecules, indicating a complex packing arrangement that contributes to the overall structural stability (Figure S19, Supporting Information). This structural motif is consistent with the formation of a highly ordered supramolecular architecture driven by directional non‐covalent interactions. Notably, the **Nap‐wfy** molecules engage in extensive intermolecular *π–π* stacking interactions with neighboring molecules, which play a critical role in stabilizing the 3D crystal lattice (**Figure** **3**D). In the crystallographic *a*‐direction, the aromatic w moiety of one molecule aligns face‐to‐face with the f side chain of an adjacent molecule, forming strong *π–π* stacking interactions with an interplanar distance of 3.7 Å, indicative of favorable orbital overlap and electronic delocalization (Figure 3A). This interaction is characteristic of aromatic‐aromatic contacts commonly observed in peptide‐based crystalline materials and contributes significantly to the directional cohesion along this axis. In the *b*‐direction, the Nap groups from neighboring molecules exhibit a well‐ordered, parallel stacking arrangement with a consistent interplanar distance of 3.8 Å (Figure 3B). This regular packing not only enhances the structural rigidity but also facilitates efficient charge transport pathways, which are essential for potential applications in organic electronics or piezoelectric devices. The naphthalene‐naphthalene stacking is further stabilized by van der Waals forces and hydrophobic effects, reinforcing the integrity of the supramolecular framework. Furthermore, along the *c*‐direction, the y moiety within one‐unit cell participates in long‐range *π–π* interactions with the w residue of a molecule in an adjacent unit cell, maintaining a distance of 3.7 Å (Figure 3C). This inter‐unit‐cell interaction extends the supramolecular network beyond the immediate coordination sphere, promoting long‐range order and mechanical stability. Collectively, the triclinic crystal structure of **Nap‐wfy** is stabilized by a 3D network of intermolecular interactions, including *π–π* stacking between the aromatic w and f side chains, Nap‐Nap stacking along the *b*‐axis, and y‐w cross‐unit‐cell π‐interactions along the *c*‐axis. These synergistic interactions not only define the molecular packing but also underpin the material's structural robustness and functional potential. The precise spatial arrangement of aromatic residues enables efficient electronic coupling, making **Nap‐wfy** a promising candidate for the rational design of functional peptide‐based crystalline materials with mechanical properties.

### 2.3 Piezoelectric Properties of Nap‐wfy Crystals

**Figure 4:**
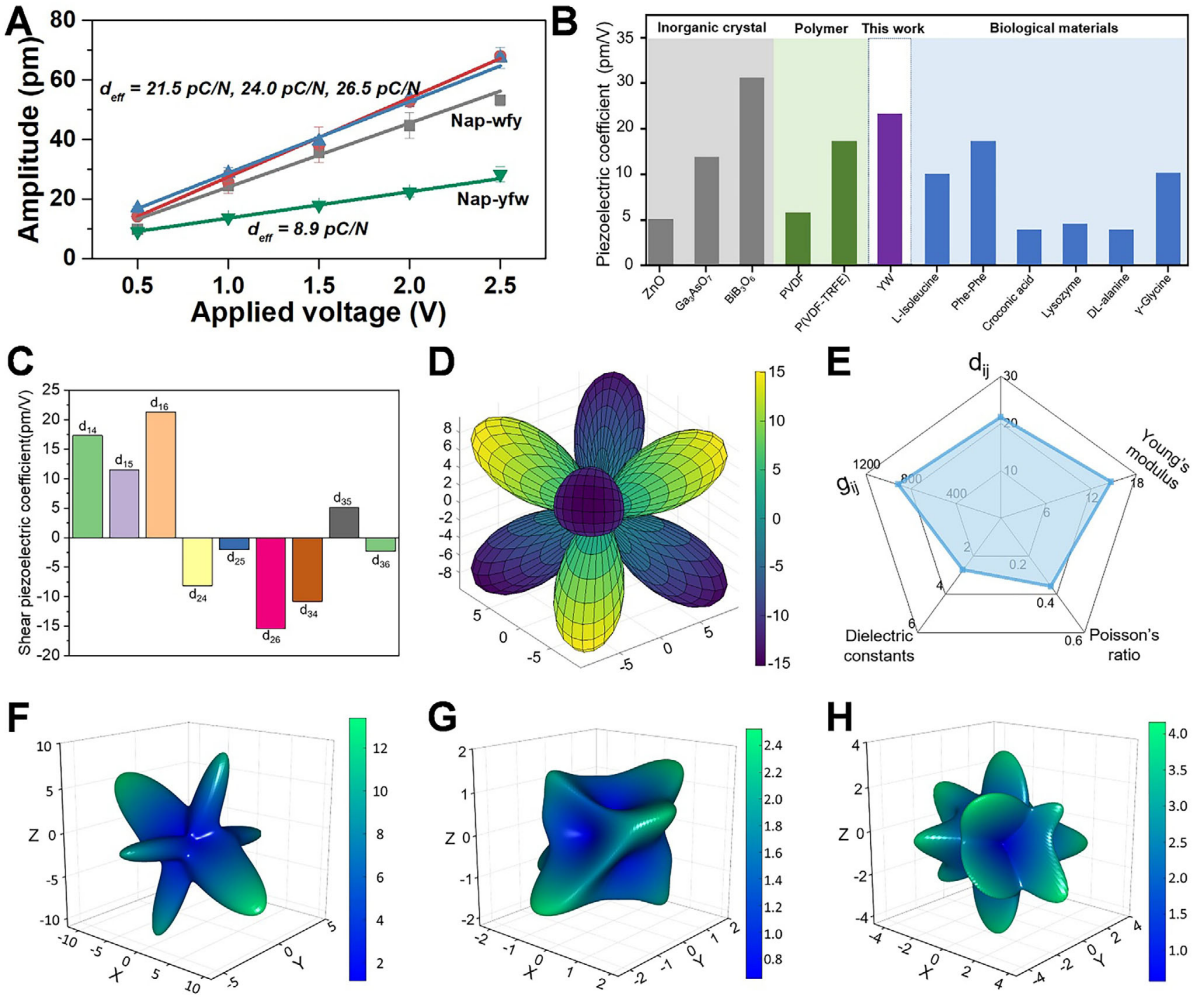
A) The derived amplitude excitation curve for the **Nap‐wfy** crystal by the PFM tip. B) Piezoelectric coefficient of **Nap‐wfy** compared with some classical inorganic crystals, polymers, and biological materials. C) Predicted Shear piezoelectric coefficient of **Nap‐wfy** crystal. D) 3D surface plot of computed piezoelectric coefficients of **Nap‐wfy** crystal. E) The comprehensive performance of **Nap‐wfy** crystal includes: the piezoelectric coefficient *d_ij_* (pC/N), Young's modulus, Poisson's ratio, dielectric constant, and piezoelectric voltage constants *g_ij_* (mVm/N). F–H) 3D plots of elastic modulus (GPa) of **Nap‐wfy** crystal: F) Directional Young's modulus, G) Spatial dependence of average shear modulus, H) Spatial dependence of shear modulus MAX.

Building upon the established knowledge regarding the morphology, secondary structure, and molecular packing of **Nap‐yfw** and **Nap‐wfy**, we employed PFM to explore the piezoelectric response of these crystals. By applying a voltage to the prepared crystals, we were able to observe their displacement, allowing us to calculate the *d_eff_* values for **Nap‐yfw** and **Nap‐wfy** crystals as 8.9 and 24.0 pC N^−1^, respectively (**Figure** **4**A). The enhanced piezoelectric performance of **Nap‐wfy** stems from a synergistic enhancement of dipole moments, driven by specific molecular and supramolecular structural features. DFT calculations confirm that the strategic position of the high‐electron density tryptophan indole ring results in a molecular dipole moment of 4.9 Debye for **Nap‐wfy**, which is greater than 3.4 Debye for **Nap‐yfw** (Figure S20, Supporting Information). Moreover, **Nap‐wfy** molecular dipoles are organized into a highly aligned packing structure along the *b*‐axis (Figure 3D), culminating in a powerful net crystal dipole moment of 7.6 Debye. This directional lattice polarization is the key factor responsible for the efficient electromechanical coupling. Notably, the d*_eff_* value obtained for the **Nap‐wfy** crystals surpassed those previously reported for biomaterials such as Pro‐Phe‐Phe (3.1 pC N^−1^), and γ‐Glycine (9.9 pC N^−1^), as well as inorganic materials such as ZnO (9.9 pC N^−1^) (Figure 4B; Table S4, Supporting Information). This remarkable finding aroused our interest, prompting us to further investigate whether the measured piezoelectric response of **Nap‐wfy** crystals aligns with the theoretically predicted value. To address this, we employed DFT calculations to predict the piezoelectric properties of crystals,^[^ ^56^, ^57^, ^58^, ^59^ ^]^ including elastic stiffness constant, piezoelectric strain constant, and charge tensor constant. Through these calculations, we obtained valuable insights into the electromechanical properties of **Nap‐wfy** crystals. The average charge tensor, voltage tensor, and stiffness tensor of **Nap‐wfy** crystals were found to exhibit a range of variation, with values spanning from −0.045 to 0.020 C m^−^^2^, −677 to 912 mVm N^−1^, and −2.367 to 21.333 GPa, respectively (Table S5, Supporting Information). Furthermore, we conducted d*_ij_* = e*_ij_*/C*_ij_* calculations to determine the piezoelectric tensor component of the crystals along different directions. The results show that the components range from 2.3 to 21.3 pC N^−1^, with the d*_16_* component being the largest (Figure 4C). The d*_16_* component was found to be the largest among them and possibly contributing the most to the experimentally measured response. Moreover, the piezoelectric coefficients of the **Nap‐wfy** crystals showed a clear orientation dependence (Figure 4D). To estimate the potential energy harvesting capability, we further calculated the dielectric constants (ε*_r_*) and piezoelectric voltage constants (g*_ij_*) of **Nap‐wfy** crystals (Table S5, Supporting Information). The relatively low dielectric constant (ε*_r_* ≈4.00) of **Nap‐wfy** crystals drives a large voltage constant g_14_ of 912 mV m N^−1^, which is larger than the values of typical inorganic materials PbTiO_3_ and BaTiO_3_.^[^ ^60^, ^61^ ^]^ These results suggest that **Nap‐wfy** crystals show great potential for sensors and piezoelectric energy harvesting. Figure 4E summarizes several performance metrics of **Nap‐wfy** crystal, including the piezoelectric coefficient d*_ij_*, Young's modulus, Poisson's ratio, dielectric constant, and piezoelectric voltage constants g*_ij_*. To gain a more intuitive understanding of elastic properties, we visualized the directional Young's modulus, which shows material's uniaxial stiffness across different orientations (Figure 4F). The directional Young's modulus exhibits significant anisotropy, with the highest stiffness values (12 GPa) found within the plane that is elliptical and oriented perpendicular to the *Z*‐axis. In addition, the shear modulus AVE and MAX surface exhibit multiple extreme points to form the shell structure of the shear modulus (Figure 4G,H). This unique mechanical property might be a key factor behind the substantial piezoelectric coefficient observed in **Nap‐wfy** crystals. Based on the combined analysis of our experimental results and theoretical calculations, we propose that the high piezoelectric coefficient exhibited by **Nap‐wfy** crystals can be primarily attributed to their highly asymmetric nature. This inherent property within the crystal structure contributes significantly to the observed high piezoelectric response.

### 2.4 Fabrication of Piezoelectric Device using Nap‐wfy Crystals

**Figure 5:**
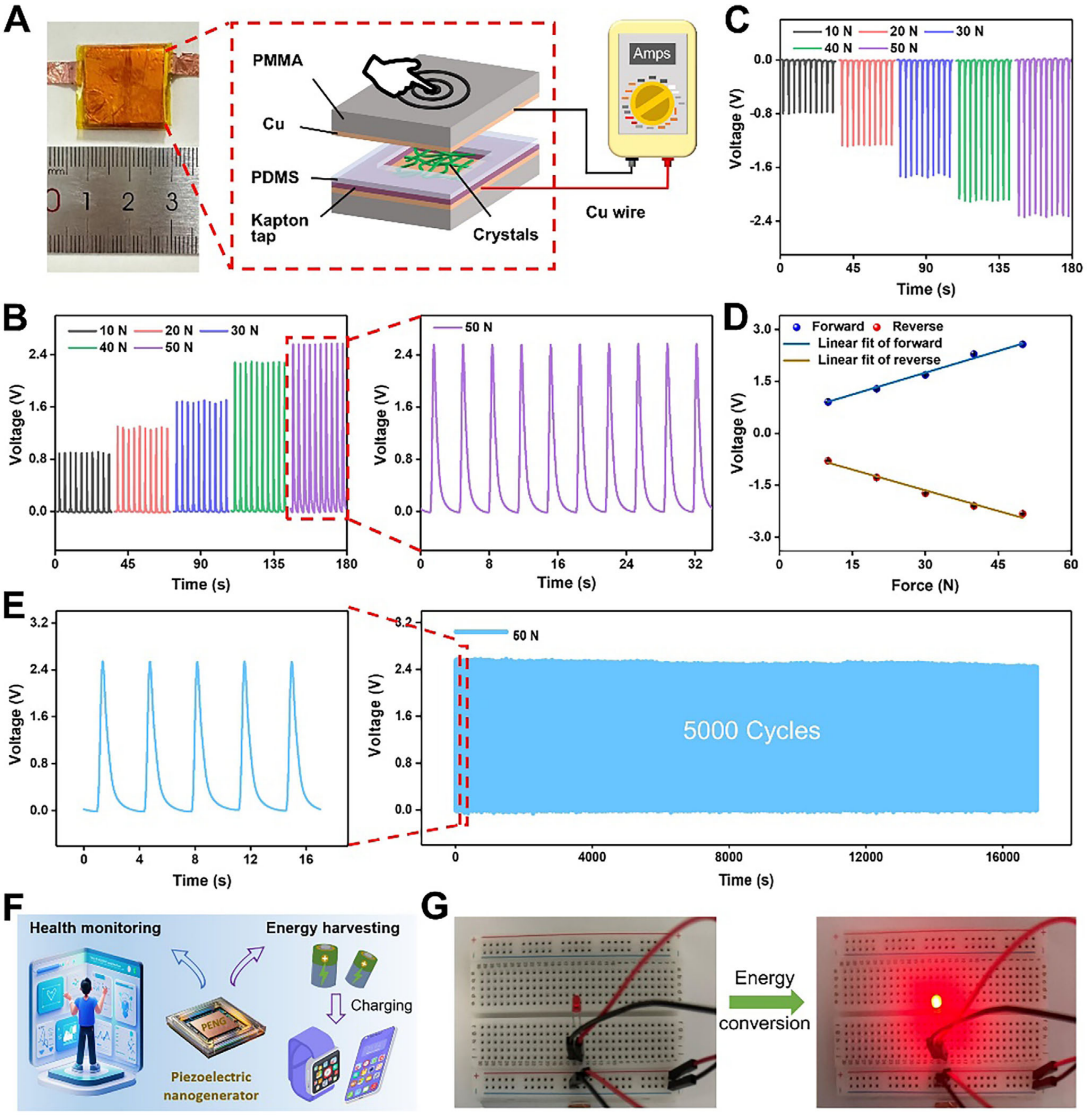
Characterization of **Nap‐wfy**‐based nanogenerator. A) Schematic illustration of the sandwiched structure comprising the **Nap‐wfy** crystals as the active component. B,C) Open‐circuit voltages of a piezoelectric nanogenerator comprising **Nap‐wfy** crystals as the active component in the (B) forward and (C) reverse connections under different applied forces. D) Linear fitting of the open‐circuit voltage output and applied pressure of the nanogenerator in the forward and reverse connections. E) Stability of the piezoelectric nanogenerator based on **Nap‐wfy** crystals over time. F) Schematic diagram of the application for energy harvesting and health monitoring. G) **Nap‐wfy**‐based piezoelectric nanogenerators illuminate LEDs by harvesting mechanical energy.

Based on the aforementioned findings, we investigated the potential of **Nap‐wfy** crystals for piezoelectric power generation. We utilized these crystals as the piezoelectric active component in the fabrication of sandwiched nanogenerators (**Figure** **5**A). During the experimental setup, two copper‐coated polymethyl methacrylate (PMMA) substrates were selected as electrodes, with the **Nap‐wfy** crystal powder sandwiched between them. To prevent damage from mechanical stress, dust, and moisture, the piezoelectric device components were hermetically sealed. Subsequently, a linear motor was employed to apply vertical pressure onto the device, and the resulting electrical signals generated by the nanogenerator were accurately measured and recorded. A range of forces, varying from 10 to 50 N, was systematically applied to the **Nap‐wfy** crystal‐based nanogenerator using a linear moto. Subsequently, the corresponding electrical signals were generated and analyzed. Remarkably, as the applied force increased from 10, 20, 30, 40, to 50 N, the nanogenerator exhibited a progressive enhancement in open‐circuit voltage, yielding values of 0.90, 1.29, 1.69, 2.29, and 2.57 V, respectively (Figure 5B; Figure S21, Supporting Information). Furthermore, we explored the impact of reversing the connection mode. When the connection mode was reversed, under applied forces varied from 10 to 50 N, the measured open‐circuit voltages were determined to be −0.80, −1.28, −1.73, −2.10, and −2.33 V, respectively (Figure 5C; Figure S22, Supporting Information). Notably, the open‐circuit voltage values also exhibited a consistent trend in the reverse connection mode, indicating remarkable electromechanical response and repeatability. Moreover, we observed a strong linear relationship between the open‐circuit voltage and the applied mechanical forces. The forward connection mode displayed a linear slope of 0.042 V N^−1^, while the reverse connection mode was −0.040 V N^−1^ (Figure 5D). This notable voltage output of 2.57 V surpasses the performance of most previously reported peptide‐based nanogenerators, such as Pro‐Phe‐Phe crystals (1.4 V),^[^ ^47^ ^]^ FF peptide microrods (1.4 V),^[^ ^52^ ^]^ cyclo‐GW crystals (1.2 V),^[^ ^62^ ^]^ and inorganic materials, such as MoS_2_ shells arrays (1.2 V),^[^ ^63^ ^]^ lead‐free LiNbO_3_ nanowires (0.46 V).^[^ ^64^ ^]^ This high piezoelectric response is likely attributed to the highly asymmetrical arrangement within the crystals, coupled with the abundance of crystals within the nanogenerator system. Additionally, we conducted short‐circuit current measurements to further evaluate the performance of the nanogenerator. When subjected to mechanical forces ranging from 10 to 50 N, the forward connection configuration generated short‐circuit currents of 2.24, 2.63, 3.22, 3.99, and 4.48 nA, respectively (Figure S23, Supporting Information). Conversely, under the same mechanical forces, the reverse connection configuration yielded short‐circuit currents of −2.41, −3.17, −3.60, −4.23, and −4.91 nA, respectively (Figure S24, Supporting Information). Similarly, the outputs of the short‐circuit current displayed a clear linear relationship with the applied mechanical forces. The corresponding slopes were determined to be 0.060 nA N^−1^ for the forward connection and −0.059 nA N^−1^ for the reverse connection. Specifically, the electrical signal outputs obtained in the forward connection mode were comparable to those measured in the reverse connection mode. This observation suggests that the generated electrical signals primarily originated from the **Nap‐wfy** crystals themselves, rather than from parasitic capacitance or contact resistance. The power output performance of the **Nap‐wfy** crystal‐based piezoelectric nanogenerators was investigated by measuring the output voltage characteristics under various external resistive loads (Figure S25, Supporting Information). The device delivered a maximum power density of 0.475 µW cm^−2^, which was comparable to existing biomolecular‐based piezoelectric generators such as Chitin,^[^ ^65^ ^]^ D‐BIO,^[^ ^66^ ^]^ and diphenylalanine,^[^ ^67^ ^]^ as well as hybrid piezoelectric systems^[^ ^68^ ^]^ such as MXene/PVDF^[^ ^69^ ^]^ and [Cu(L‐phe)(Bpy)]/PVDF^[^ ^70^ ^]^ (Table S6, Supporting Information). This desirable power density suggests its potential for use in electromechanical energy conversion. In addition, the practical charge storage performance of the **Nap‐wfy**‐based piezoelectric nanogenerators was further demonstrated by charging external capacitors (Figure S26, Supporting Information). The device successfully charged a 0.1 µF capacitor to over 18 V within minutes, demonstrating robust charge retention and direct energy storage functionality. Moreover, we tested the stable power generation performance of the device at a cyclic force of 50 N. The obtained voltage showed no obvious attenuation during 5000 pressing‐releasing cycles (Figure 5E), indicating the high durability of the **Nap‐wfy** crystal‐based device. In addition, **Nap‐wfy** crystals retained their structural integrity and exhibited nearly identical morphological patterns after immersion in water for 7 days (Figure S27, Supporting Information), demonstrating their excellent water‐resistant stability and robustness under prolonged aqueous exposure. To evaluate the environmental stability of our devices, the performance of the **Nap‐wfy**‐based piezoelectric nanogenerators was characterized under high‐humidity conditions. As shown in Figure S28 (Supporting Information), the devices exhibited excellent stability when maintained at a relative humidity of ≈90% for 12 days, with no significant degradation in voltage output.

These compelling results provide robust evidence for the exceptional capability of **Nap‐wfy** crystals as effective nanogenerators in piezoelectric energy harvesting, and the demonstrated linear relationship between the open‐circuit voltage/current and applied mechanical forces highlights the reliability of nanogenerators. Moreover, the consistent performance in both connection modes verifies the genuine contribution of the **Nap‐wfy** crystals in generating electrical signals, affirming their suitability for practical energy conversion applications. Finally, we demonstrated that the piezoelectric device can convert the harvested mechanical energy into electric energy and could drive an LED (Figure 5F,G; Movie S1, Supporting Information). Therefore, these results confirm **Nap‐wfy** crystals‐based nanogenerator can realize the efficient conversion between mechanical force and electric energy.

## 3 Discussion

In summary, our study successfully achieved the generation of various types of anisotropic crystals as well as isotropic hydrogels by simply adjusting the position of the amino acid within the tripeptides. Specifically, when the middle amino acid was f, we observed the formation of anisotropic crystals exhibiting superior piezoelectric properties. The microED technique confirmed the presence of supramolecular interactions and the packing arrangement of amino acids, with the molecular packing controlled by both intermolecular and intramolecular *π–π* stacking interactions. PFM measurements and DFT calculations were performed to further elucidate the piezoelectric property of **Nap‐wfy** crystals. These analyses provided additional evidence supporting the presence of piezoelectricity in **Nap‐wfy** crystals, primarily attributed to the inherent low symmetry within the supramolecular packing arrangement. Moreover, we successfully demonstrated the practical application of **Nap‐wfy** crystals by fabricating nanogenerators capable of piezoelectric power generation. Remarkably, these nanogenerators exhibited an output voltage of 2.57 V when subjected to a mechanical force of 50 N. The significance of this work lies in the development of an efficient strategy for fabricating peptide‐based supramolecular structures with inherent piezoelectric properties, and the fabricated nanogenerators could serve as promising energy harvesting devices, effectively converting mechanical forces into electrical energy, which could contribute to the advancement of materials science and offer potential avenues for the design and development of novel peptide‐based piezoelectric materials for various applications.

## 4 Experimental Section

### Experiment Materials

All reagents used were of analytical grade and were used as received without any further purification. All Fmoc‐protected amino acids were purchased from GL Biochem Ltd. (China). Piperidine was received from Sinopharm Chemical Reagent Co., Ltd. (China). N, N‐diisopropylethylamine (DIPEA) was purchased from Aladdin Biochemical Technology Co., Ltd. (China). 2‐(1H‐Benzotriazole‐1‐yl)‐1,1,3,3‐tetramethyluronium hexafluorophosphate (HBTU) was purchased from GL Biochem Ltd. (China).

### Synthesis of Tripeptide

The tripeptides investigated in this manuscript were synthesized using the SPPS method. The synthesis process involved the utilization of 2‐chlorotrityl chloride resin (Hecheng Science & Technology CO., Ltd., China) and Fmoc‐protected amino acids. To initiate the synthesis, the resin was first swollen in dry DCM for 15 min. Following this, the first amino acid was added and allowed to react with the resin at its C‐terminus for 1 h. To quench unreacted sites on the resin, a blocking solution (MeOH: DIPEA: DCM = 2:1:17) was introduced. The Fmoc protecting group was then removed from the resin by treating it with a solution of 20% piperidine in DMF for 30 min. The resin was subsequently washed with DMF for six times. This process was repeated for the addition of the other two amino acids and 2‐naphthylacetic acid, with the resin being washed with DMF after each step and the Fmoc group being removed using 20% piperidine in DMF solution. In the final step, the resin was washed with DMF and DCM for six times, respectively, and the peptide was cleaved from the resin by treating it with a cocktail solution of trifluoroacetic acid (TFA): triisopropylsilane (TIS): water (95%: 2.5%: 2.5%) for 0.5 h. The cleaved peptide was then evaporated under vacuum and precipitated in cold diethyl ether. The resulting powder was collected and subjected to purification using HPLC equipped with a reverse‐phase C18 column (Waters, RP18 10.0 µm, 19 × 150 mm). The eluents used for HPLC purification were CH3CN (0.1% TFA) and water (0.1% TFA). The yield of each peptide was ≈90%.

### Preparation of Hydrogels and Crystals

The hydrogels and crystals were prepared using the heating‐cooling method in this study. The tripeptides were dispersed in phosphate buffer saline (PBS) solution at a concentration of 3.0 mg mL^−1^. Subsequently, the samples were incubated in an 80 °C water bath for 2 min, followed by removal and further incubation at room temperature. Remarkably, **Nap‐wyf**, **Nap‐fyw**, **Nap‐fwy**, and **Nap‐ywf** tripeptides were observed to form stable hydrogels within 2 min, while the **Nap‐yfw** and **Nap‐wfy** tripeptides rapidly formed precipitates composed of crystals within a few seconds.

### Microcrystal Electron Diffraction (MicroED)

The analysis of the single crystal structure of the **Nap‐wfy** crystal was conducted using MicroED, with the project being undertaken in collaboration with Shenzhen Jingtai Technology Co. Ltd. The crystals were carefully positioned on the TEM (Thermo Scientific Glacios) holder, and a systematic search was performed to identify the target crystal. Subsequently, the crystal was rotated at a controlled speed, allowing for the collection of diffraction images in the diffraction mode. Through the analysis of the diffraction data obtained at various rotational angles, the electron density map of the unit cell was meticulously examined. This analysis enabled the determination of the atomic coordinates within the unit cell, ultimately leading to the elucidation of the complete structure of the single crystal.

### Density Functional Theory (DFT) Calculations

Electromechanical properties were predicted from periodic density functional theory (DFT) calculations on the crystals using the VASP code. The exchange‐correlation interaction of electrons was prescribed by the PBE functional and projector augmented wave (PAW) pseudopotentials. The nonlocal van der Waals density functional, rev‐vdW‐DF2, was used to process the exchange‐correlation functional. The wave function was represented as a plane wave expansion that was truncated at a cut‐off energy of 600 eV. The convergence criterion for the total energy and atomic force components were set to 10–6 eV and 0.001 eV Å^−1^. The piezoelectric strain constants and dielectric tensors were calculated using Density Functional Perturbation Theory (DFPT).

### Fabrication of Piezoelectric Energy Harvester

In a typical sandwich‐type Nap‐wfy‐based piezoelectric energy harvester fabrication process, two 2.0 × 2.0 cm^−2^ polymethyl methacrylate (PMMA) substrates were sputtered with copper (Cu) film that served as top and bottom electrodes. **Nap‐wfy** crystal powders were filled densely into a polydimethylsiloxane (PDMS, Sylgard 184) protective layer with a hole of 1.0 × 1.0 cm^2^, which was sandwiched between the two electrodes. To ensure that there were no gaps inside the device, Kapton tape and double‐sided tape were applied between the PDMS protective layer and the electrodes. To prevent cracking of the substrate under a strong mechanical impact force from the linear motor, a PDMS damping layer was coated on the top electrode. Two Cu wires were connected to the bottom and top electrodes, respectively, which could transmit output signals to the measuring equipment. Finally, the surface of the device was tightly packed with Kapton tape.

### Power Generation

The **Nap‐wfy** crystal‐based energy harvester was vertically fixed onto a stainless‐steel plate. During the testing, a force‐controlled linear motor (LinMot PS01‐37×120‐C) was used to apply cyclic forces to periodically press the energy harvester, and the driving force applied to the energy harvester was measured with a force sensor. The outputs of the energy harvester were collected using an electrometer (Keithley 6514) and a data acquisition system (NI USB‐6218).

## Conflict of Interest

The authors declare no conflict of interest.

## Supporting information



Supporting Information

Supporting Information

## Data Availability

The data that support the findings of this study are available from the corresponding author upon reasonable request.
