# Supplementary material for: Peptide Sequence Programmed Piezoelectric Response by Supramolecular Self‐Assembly
Source: Adv Sci (Weinh). 2025 Dec 15;13(12):e15237. doi: 10.1002/advs.202515237 (PMC12948212; doi:10.1002/advs.202515237)
Supplement: Supplementary file 1 — Supporting Information [file ADVS-13-e15237-s001.docx]

Supplementary Materials for

**Peptide Sequence Programmed Piezoelectric Response by Supramolecular Self-Assembly**

Xuejiao Yang, Shuaijie Liu, Honglei Lu, Yuehui Wang, Hongyue Zhang, Wei Ji,* Huaimin Wang*

**Email:** weiji@cqu.edu.cn (W. J.); wanghuaimin@westlake.edu.cn (H.M.W.)

**Content**

**S1. Characterizations**..…………………...........................................................…............3

**S2. Supporting figures**…………………………….….................................………............5

**Figure S1.** ^1^H NMR spectrum of **Nap-fyw**.

**Figure S2.** ^1^H NMR spectrum of **Nap-fwy**.

**Figure S3.** ^1^H NMR spectrum of **Nap-ywf**.

**Figure S4.** ^1^H NMR spectrum of **Nap-yfw**.

**Figure S5.** ^1^H NMR spectrum of **Nap-wfy**.

**Figure S6.** LC-MS of A) **Nap-fyw**, B) **Nap-fwy**, C) **Nap-ywf**, D) **Nap-yfw**, E) **Nap-wfy**, F) yfw, and G) wfy, respectively.

**Figure S7.** Optical photos of nanostructures formed by tripeptides through “heating-cooling” strategy.

**Figure S8.** ^1^H NMR spectrum of **yfw**.

**Figure S9.** ^1^H NMR spectrum of **wfy**.

**Figure S10.** A) Optical photos and B, C) TEM images of nanostructures formed by B) **yfw** and C) **wfy** through “heating-cooling” strategy.

**Figure S11.** TEM images of nanostructures formed by tripeptides.

**Figure S12.** SEM images of nanostructures formed by tripeptides. The concentration of tripeptide is 3.0 mg/mL.

**Figure S13.** A-F) SEM images, statistic G, I) length and H, J) diameter of crystals formed by A-C, G, H) **Nap-yfw** and D-F, I, J) **Nap-wfy**, respectively.

**Figure S14.** AFM images of crystals formed by **Nap-yfw**.

**Figure S15.** A-C) CD and D-F) FTTR spectra of nanostructures formed by tripeptides.

**Figure S16.** Congo red staining with A-D) hydrogels and E) crystals formed by tripeptides.

**Figure S17.** PXRD spectra of A, B) hydrogels and C) crystals formed by tripeptides.

**Figure S18.** TGA analysis of **Nap-yfw** crystals.

**Figure S19.** The unit cell of **Nap-wfy** crystals.

**Figure S20.** Molecule dipole moment of A) **Nap-wfy** and B) **Nap-yfw**.

**Figure S21.** A-E) Forward open-circuit voltage output obtained from the piezoelectric energy harvester based on pure **Nap-wfy** crystals under various applied forces, A) 10 N, B) 20 N, C) 30 N, D) 40 N, and E) 50 N. F) Linear dependence of the voltage output on the applied force.

**Figure S22.** A-E) Reverse open-circuit voltage output obtained from the piezoelectric energy harvester based on pure **Nap-wfy** crystals under various applied forces, A) 10 N, B) 20 N, C) 30 N, D) 40 N, and E) 50 N. F) Linear dependence of the voltage output on the applied force.

**Figure S23.** A-E) Forward short-circuit current output obtained from the piezoelectric energy harvester based on pure **Nap-wfy** crystals under various applied forces, A) 10 N, B) 20 N, C) 30 N, D) 40 N, and E) 50 N. F) Linear dependence of the current output on the applied force.

**Figure S24.** A-E) Reverse short-circuit current output obtained from the piezoelectric energy harvester based on pure **Nap-wfy** crystals under various applied forces, A) 10 N, B) 20 N, C) 30 N, D) 40 N, and E) 50 N. F) Linear dependence of the current output on the applied force.

**Figure S25.** Dependence of the power output of the **Nap-wfy** crystal-based piezoelectric nanogenerators on the resistance of the external load under 50 N compressive force.

**Figure S26.** The charging capacity of capacitors of the **Nap-wfy** crystal-based piezoelectric nanogenerators under 50 N compressive force.

**Figure S27.** Optical microscope images of **Nap-wfy** crystals upon immersion in water for 7 days.

**Figure S28.** The voltage output of the **Nap-wfy**-based piezoelectric nanogenerators with 50 N force under relative humidity of around 90% for 1 day, 6 days and 12 days.

**S3. Supporting tables**…………………………….…...............................……….............18

**Table S1.** Single-crystal structure of **Nap-wfy** crystals determined by MicroED.

**Table S2.** Atomic fractional coordinate x, y, z (Å) and equivalent isotropic displacement parameters U_eq_ (Å^2^) inside **Nap-wfy** single crystal.

**Table S3.** Atomic anisotropic displacement parameters (Å^2^) inside **Nap-wfy** single crystal.

**Table S4.** Piezoelectric materials and their maximum piezoelectric response.

**Table S5.** Predicted piezoelectric property of **Nap-wfy** crystals, including maximum piezoelectric stress constants e*_ij_* (C/m^2^), maximum piezoelectric strain constants d*_ij_* (pC/N), maximum piezoelectric voltage constants g*_ij_* (mVm/N), and minimum elastic constants C*_ij_* (GPa).

**Table S6.** Comparison of power outputs of piezoelectric nanogenerators based on different materials.

**S1. Characterizations**

**S1.1 Nuclear magnetic resonance spectroscopy (NMR)**

The ^1^H NMR was recorded on 500 M AVANCE NEO spectrometer (Bruker, US), referenced to Si(CH_3_)_4_. The solvent used in the measurement was *d_6_*-DMSO.

**S1.2 Liquid chromatography-mass spectrometer (LC-MS)**

The synthesized peptides were dissolved in MeOH at a concentration of 0.2 mg mL^-1^, then the purity and molecular weight of samples were detected using LC-MS (Agilent 1260 Infinity) equipped with C18 column, the mobile phase was acetonitrile (0.5‰ TFA) and deionized water (0.5‰ TFA).

**S1.3 Optical microscope**

The crystals were deposited onto a glass slide to facilitate their characterization, and the morphologies of the crystals were assessed using an EVos FL Auto 2 microscope (Thermo Fisher Scientific, US).

**S1.4 Birefringent microscope**

Following the preparation of the hydrogels and crystals, we meticulously dispersed the nanostructures onto a glass slide. Subsequently, the nanostructures were imaged using the Oosight Imaging System (Hamilton Thorne, US), which is equipped with polarization optics and a differential interference contrast (DIC) module, integrated into the Olympus IX73 microscope (Japan).

**S1.5 Scanning electron microscope (SEM)**

A 100 μL aliquot of hydrogels or crystals was carefully deposited onto a thin glass sheet, then the excess samples were subsequently removed using a filter paper to ensure an even distribution. To enhance the conductivity and imaging quality, a thin Pt film (5 nm) was deposited onto the crystals using an ion sputtering coating instrument (EMS/150TS, UK). The resulting prepared samples were then subjected to detailed examination using a Hitachi Field Emission Scanning Electron Microscope (Regulus 8230, Japan) operating at an acceleration voltage of 5.0 kV.

**S1.6 Transmission electron microscope (TEM)**

A 10 μL aliquot of the prepared hydrogels or crystals was meticulously deposited onto a carbon-coated copper grid with 200 meshes, then the excess samples were carefully eliminated using a filter paper to ensure a uniform distribution. To enhance the contrast and facilitate imaging, 10 μL of uranyl acetate (UA) was added to stain the samples. Subsequently, the samples were left to air dry, and the prepared samples were subjected to comprehensive investigation using a Talos L120C Transmission Electron Microscope (TEM) (Thermo Fisher, US) operating at an acceleration voltage of 120 kV.

**S1.7 Atomic force microscope (AFM) and piezoresponse force microscope (PFM)**

A 100 μL aliquot of the hydrogels or crystals was evenly dispersed onto a mica sheet, followed by a drying process utilizing nitrogen purging. The resulting dried samples were then subjected to morphological analysis using an Atomic Force Microscope (AFM) in non-contact (tapping) mode, and the AFM models employed were the Cypher ES (US) and the AIST-NT Smart AFM system. In addition, the piezoelectric coefficient of the crystals was assessed using the DART SS PFM (Dual AC resonance tracking - Single Sideband Piezoresponse Force Microscopy) mode.

**S1.8 Circular dichroism (CD) spectra**

A 100 μL aliquot of either the hydrogel or crystals was carefully introduced into a quartz cell with a 0.1 cm path length. Subsequently, the CD signal was meticulously recorded in the wavelength range of 180 nm to 340 nm. This CD analysis was conducted using a circular dichroism spectrometer (Applied Photophysics Ltd, UK).

**S1.9 Fourier Transform Infrared Spectroscopy (FTIR)**

The lyophilized hydrogels or crystals were meticulously positioned onto the attenuated total reflection (ATR) holder, ensuring proper alignment and contact, and the Fourier-transform infrared (FTIR) signals were then methodically recorded using a Nicolet iS50 FTIR spectrometer from Thermo Fisher (US).

**S1.10 Confocal laser scanning microscope (CLSM)**

In order to visualize the secondary structure of hydrogels and crystals, we incubated prepared hydrogels or crystals with Congo red (1 mM) for a duration of 30 min at room temperature, facilitating the interaction between the dye and the prepared nanostructures. Subsequently, the hydrogels and crystals were imaged using a Zeiss LSM800 Confocal Laser Scanning Microscope (CLSM, Zeiss, Germany).

**S1.11 Powder X-ray diffraction (PXRD)**

The PXRD spectra of hydrogels and crystals were investigated using a powerful X-ray diffractometer (D8 advance, Bruker, Germany) equipped with Cu Kα radiation.

**S1.12 Thermogravimetric analysis (TGA)**

The formed **Nap-wfy** crystals were subjected to freeze-drying to obtain a powdered form. Subsequently, the thermal stability of these crystals was meticulously investigated using a TGA/DSC-FTIR-GCMS system (Mettler Toledo-Thermal Fisher-Agilent, Switzerland/US) under a dry nitrogen atmosphere with a constant flow rate of 10 mL/min. To assess the thermal properties of the crystals, a heating rate of 10 K/min was employed while monitoring the temperature range of 30 ℃ to 500 ℃.

**S2. Supporting figures**


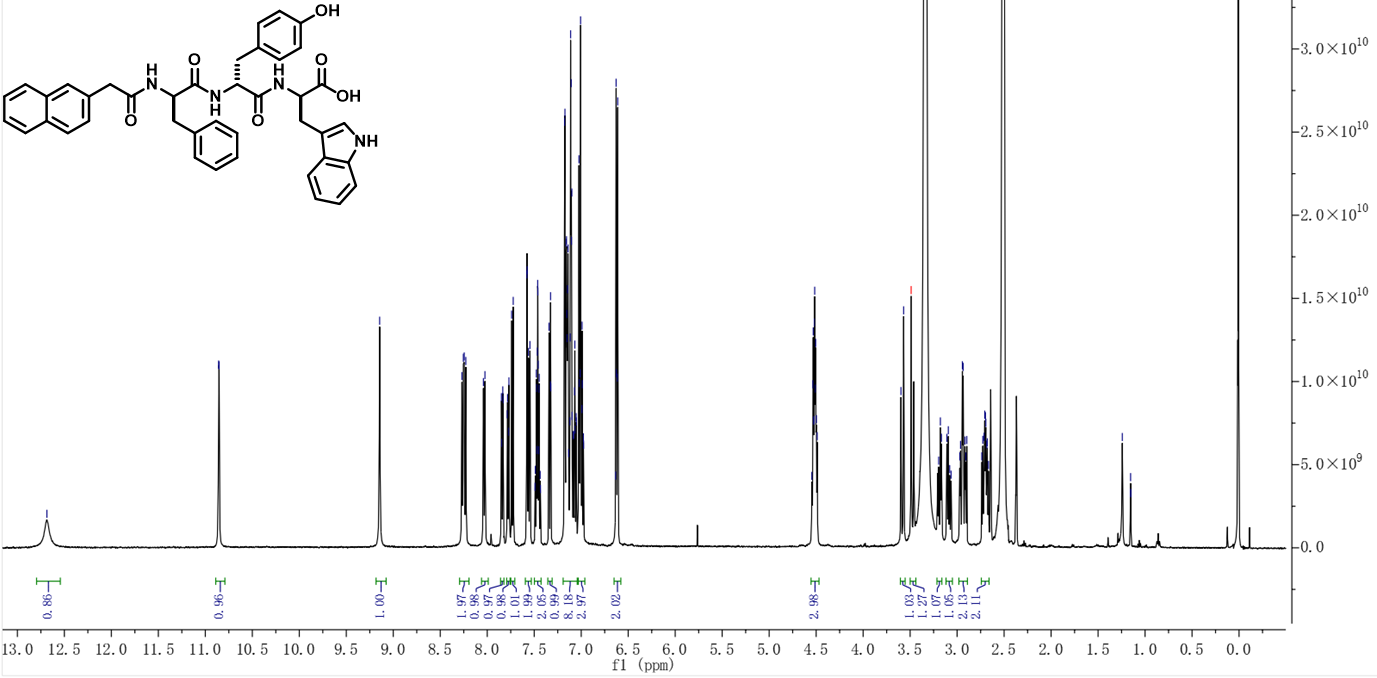


Figure S1. ^1^H NMR spectrum of Nap-fyw: δ12.7 ppm (br, 1H, COOH), δ10.8 ppm (s, 1H, NH in tryptophan), δ9.1 ppm (s, 1H, OH in tyrosine), δ8.3 ppm (m, 2H, NH), δ8.0 ppm (m, 1H, NH), δ7.9 ppm (m, 1H, CH), δ7.8 ppm (m, 1H, CH), δ7.7 ppm (m, 1H, CH), δ7.6 ppm (m, 2H, CH), δ7.4 ppm (m, 2H, CH), δ7.3 ppm (m, 1H, CH), δ7.1-7.0 ppm (m, 8H, CH), δ7.0 ppm (m, 3H, CH), δ6.6 ppm (m, 2H, CH), δ4.5 ppm (m, 3H, CH), δ3.6-3.5 ppm (m, 2H, CH_2_), δ3.2-3.0 ppm (m, 2H, CH_2_), δ2.9 ppm (m, 2H, CH_2_), δ2.7 ppm (m, 2H, CH_2_).


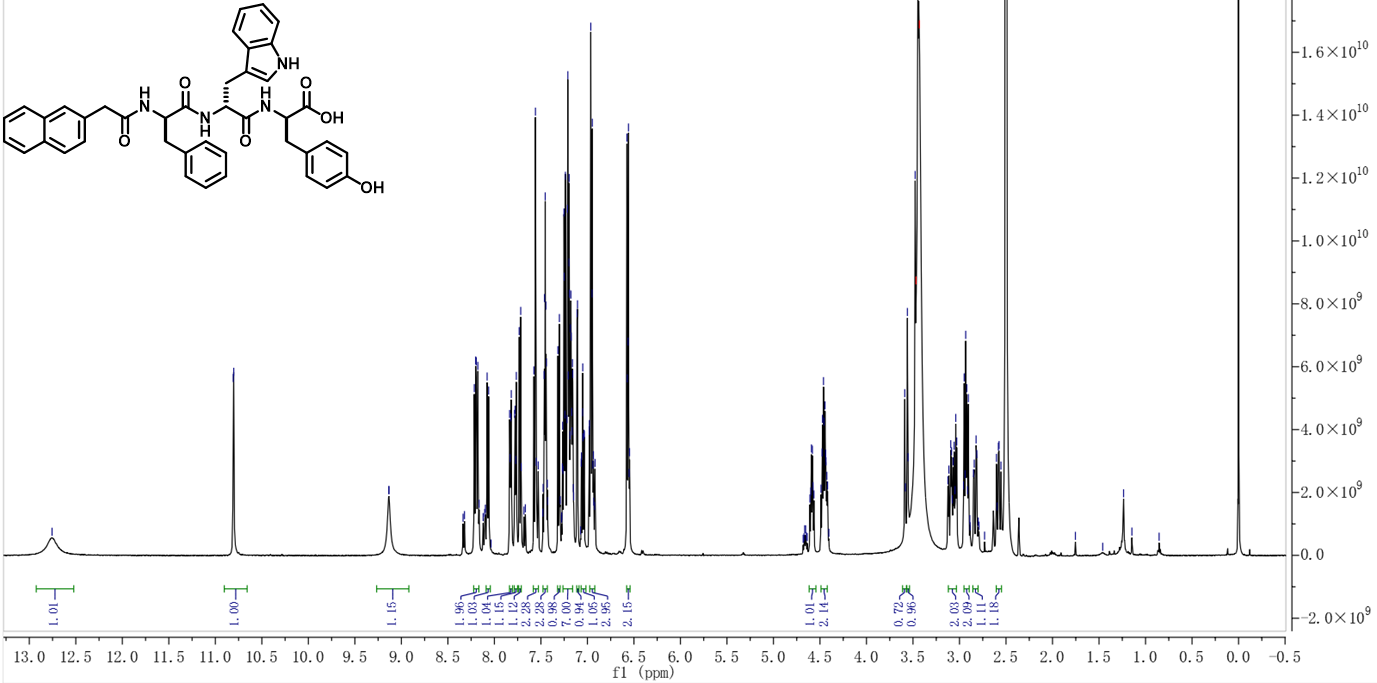


Figure S2. ^1^H NMR spectrum of Nap-fwy: δ12.7 ppm (br, 1H, COOH), δ10.8 ppm (s, 1H, NH in tryptophan), δ9.1 ppm (s, 1H, OH in tyrosine), δ8.3 ppm (m, 2H, NH), δ8.0 ppm (m, 1H, NH), δ7.9 ppm (m, 1H, CH), δ7.8 ppm (m, 1H, CH), δ7.7 ppm (m, 1H, CH), δ7.6 ppm (m, 2H, CH), δ7.4 ppm (m, 2H, CH), δ7.3 ppm (m, 1H, CH), δ7.1-7.0 ppm (m, 8H, CH), δ7.0 ppm (m, 3H, CH), δ6.6 ppm (m, 2H, CH), δ4.5 ppm (m, 3H, CH), δ3.6-3.5 ppm (m, 2H, CH_2_), δ3.2-3.0 ppm (m, 2H, CH_2_), δ2.9 ppm (m, 2H, CH_2_), δ2.7 ppm (m, 2H, CH_2_).


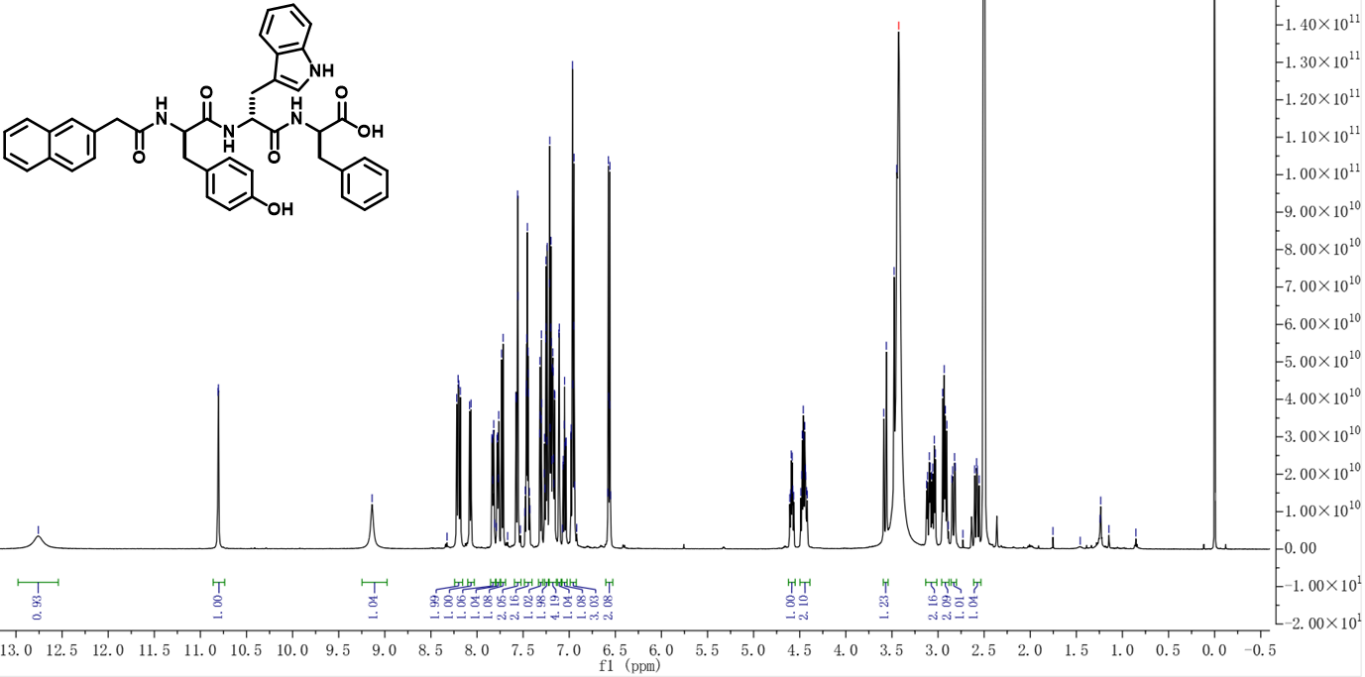


Figure S3. ^1^H NMR spectrum of Nap-ywf: δ12.7 ppm (br, 1H, COOH), δ10.8 ppm (s, 1H, NH in tryptophan), δ9.1 ppm (s, 1H, OH in tyrosine), δ8.2 ppm (m, 2H, NH), δ8.1 ppm (m, 1H, NH), δ7.9 ppm (m, 1H, CH), δ7.8 ppm (m, 1H, CH), δ7.7 ppm (m, 1H, CH), δ7.6 ppm (m, 2H, CH), δ7.5 ppm (m, 2H, CH), δ7.3 ppm (m, 1H, CH), δ7.2 ppm (m, 2H, CH), δ7.2 ppm (m, 4H, CH), δ7.1 ppm (m, 1H, CH), δ7.0 ppm (m, 1H, CH), δ6.9 ppm (m, 3H, CH), δ6.6 ppm (m, 2H, CH), δ4.5 ppm (m, 1H, CH), δ4.4 ppm (m, 2H, CH), δ3.5 ppm (m, 2H, CH_2_), δ3.1 ppm (m, 2H, CH_2_), δ2.9 ppm (m, 2H, CH_2_), δ2.8 ppm (m, 1H, CH in CH_2_), δ2.6 ppm (m, 1H, CH in CH_2_).


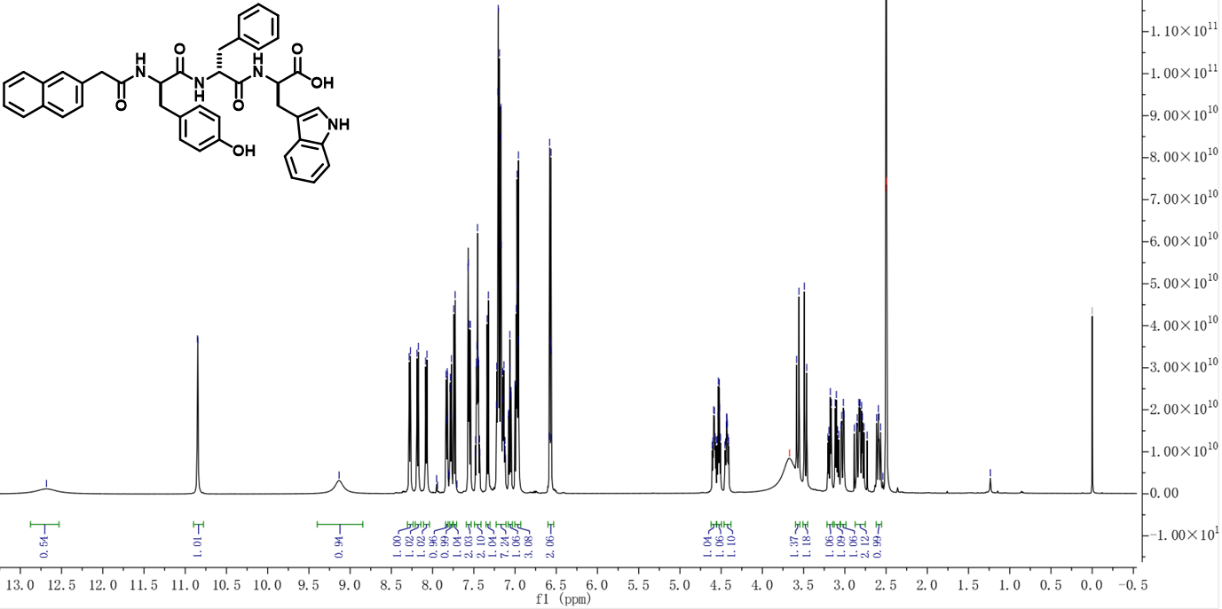


Figure S4. ^1^H NMR spectrum of Nap-yfw: δ12.6 ppm (br, 1H, COOH), δ10.8 ppm (s, 1H, NH in tryptophan), δ9.1 ppm (s, 1H, OH in tyrosine), δ8.3 ppm (m, 1H, NH), δ8.2 ppm (m, 1H, NH), δ8.1 ppm (m, 1H, NH), δ7.9 ppm (m, 1H, CH), δ7.8 ppm (m, 1H, CH), δ7.7 ppm (m, 1H, CH), δ7.6 ppm (m, 2H, CH), δ7.4 ppm (m, 2H, CH), δ7.3 ppm (m, 1H, CH), δ7.2-7.1 ppm (m, 7H, CH), δ7.1 ppm (m, 1H, CH), δ7.0 ppm (m, 3H, CH), δ6.6 ppm (m, 2H, CH), δ4.6 ppm (m, 1H, CH), δ4.5 ppm (m, 1H, CH), δ4.4 ppm (m, 1H, CH), δ3.5-3.4 ppm (m, 2H, CH_2_), δ3.2-3.1 ppm (m, 2H, CH_2_), δ3.0 ppm (m, 1H, CH_2_), δ2.8 ppm (m, 2H, CH_2_), δ2.5 ppm (m, 1H, CH_2_).


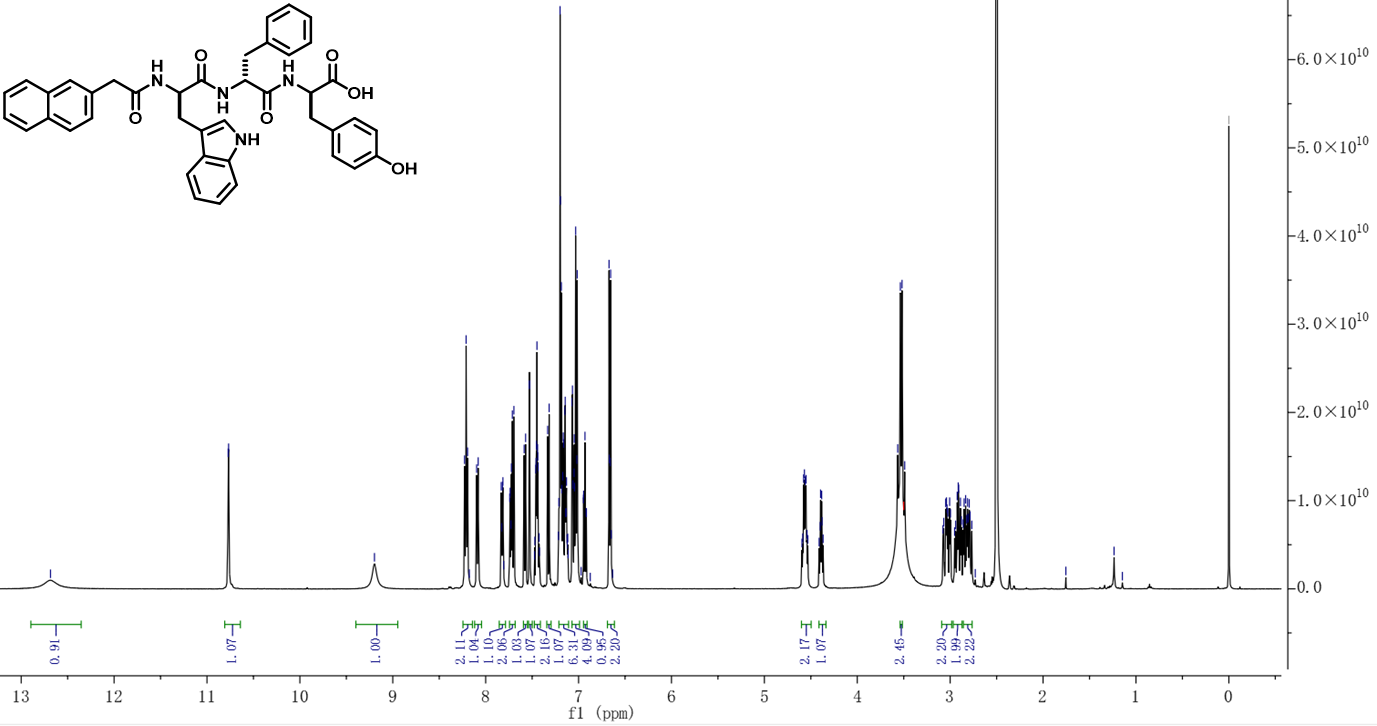


Figure S5. ^1^H NMR spectrum of Nap-wfy: δ12.7 ppm (br, 1H, COOH), δ10.7 ppm (s, 1H, NH in tryptophan), δ9.2 ppm (s, 1H, OH in tyrosine), δ8.2 ppm (m, 2H, NH), δ8.1 ppm (m, 1H, NH), δ7.8 ppm (m, 1H, CH), δ7.7 ppm (m, 2H, CH), δ7.6 ppm (m, 1H, CH), δ7.5 ppm (m, 1H, CH), δ7.4 ppm (m, 2H, CH), δ7.3 ppm (m, 1H, CH), δ7.2-7.1 ppm (m, 6H, CH), δ7.0-6.9 ppm (m, 5H, CH), δ6.6 ppm (m, 2H, CH), δ4.5 ppm (m, 2H, CH), δ4.4 ppm (m, 1H, CH), δ3.5 ppm (m, 2H, CH_2_), δ3.0 ppm (m, 2H, CH_2_), δ2.9 ppm (m, 2H, CH_2_), δ2.8 ppm (m, 2H, CH_2_).


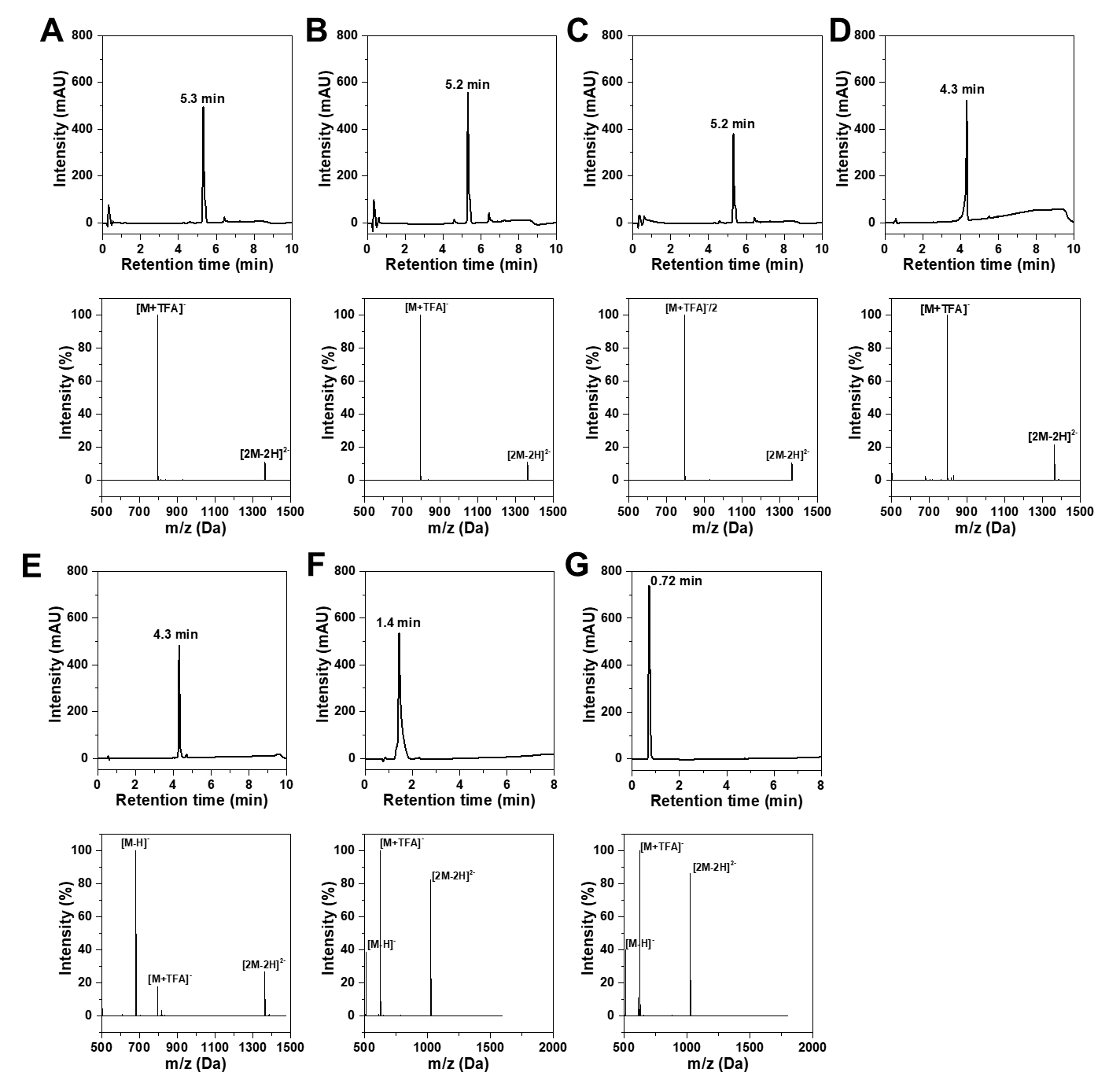


Figure S6. LC-MS of A) Nap-fyw, B) Nap-fwy, C) Nap-ywf, D) Nap-yfw, E) Nap-wfy, F) yfw, and G) wfy, respectively.


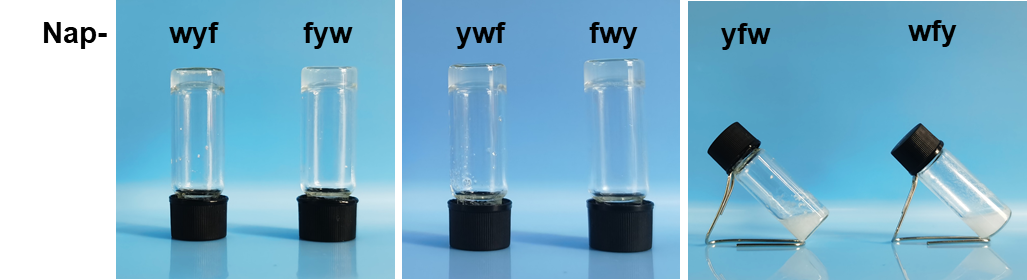


Figure S7. Optical photos of nanostructures formed by tripeptides through “heating-cooling” strategy. The concentration of tripeptide is 3.0 mg/mL.

*
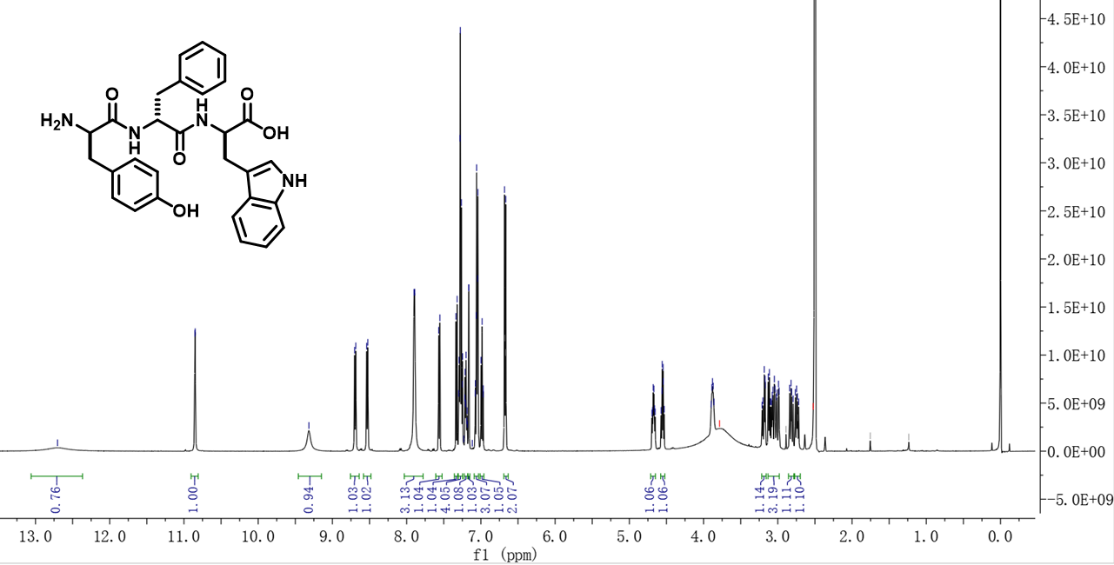
*

Figure S8. ^1^H NMR spectrum of yfw: δ12.7 ppm (br, 1H, COOH), δ10.8 ppm (s, 1H, NH in tryptophan), δ9.3 ppm (s, 1H, OH in tyrosine), δ8.7 ppm (m, 1H, NH in NH_2_), δ8.5 ppm (m, 1H, NH in NH_2_), δ7.9 ppm (m, 3H, NH), δ7.5 ppm (m, 1H, CH), δ7.3 ppm (m, 1H, CH), δ7.3 ppm (m, 4H, CH), δ7.2 ppm (m, 2H, CH), δ7.1 ppm (m, 3H, CH), δ6.9 ppm (m, 1H, CH), δ6.7 ppm (m, 2H, CH), δ4.7 ppm (m, 1H, CH), 4.5 ppm (m, 1H, CH), δ3.2-3.0 ppm (m, 4H, 2CH_2_), δ2.8-2.7 ppm (m, 2H, CH_2_).

*
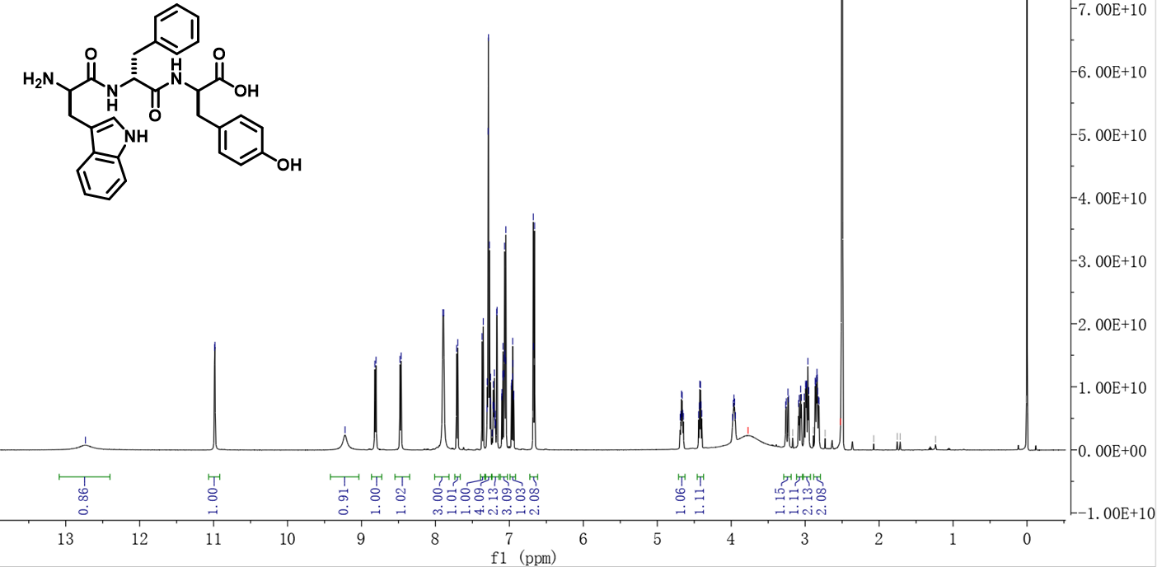
*

**Figure S9.** ^1^H NMR spectrum of **wfy**: δ12.8 ppm (br, 1H, COOH), δ11.0 ppm (s, 1H, NH in tryptophan), δ9.2 ppm (s, 1H, OH in tyrosine), δ8.8 ppm (m, 1H, NH in NH_2_), δ8.5 ppm (m, 1H, NH in NH_2_), δ7.9 ppm (m, 3H, NH), δ7.7 ppm (m, 1H, CH), δ7.4 ppm (m, 1H, CH), δ7.3 ppm (m, 4H, CH), δ7.2 ppm (m, 2H, CH), δ7.1 ppm (m, 3H, CH), δ7.0 ppm (m, 1H, CH), δ6.6 ppm (m, 2H, CH), δ4.6 ppm (m, 1H, CH), 4.4 ppm (m, 1H, CH), δ3.2-3.0 ppm (m, 2H, CH_2_), δ2.9 ppm (m, 2H, CH_2_), δ2.8 ppm (m, 2H, CH_2_).

*
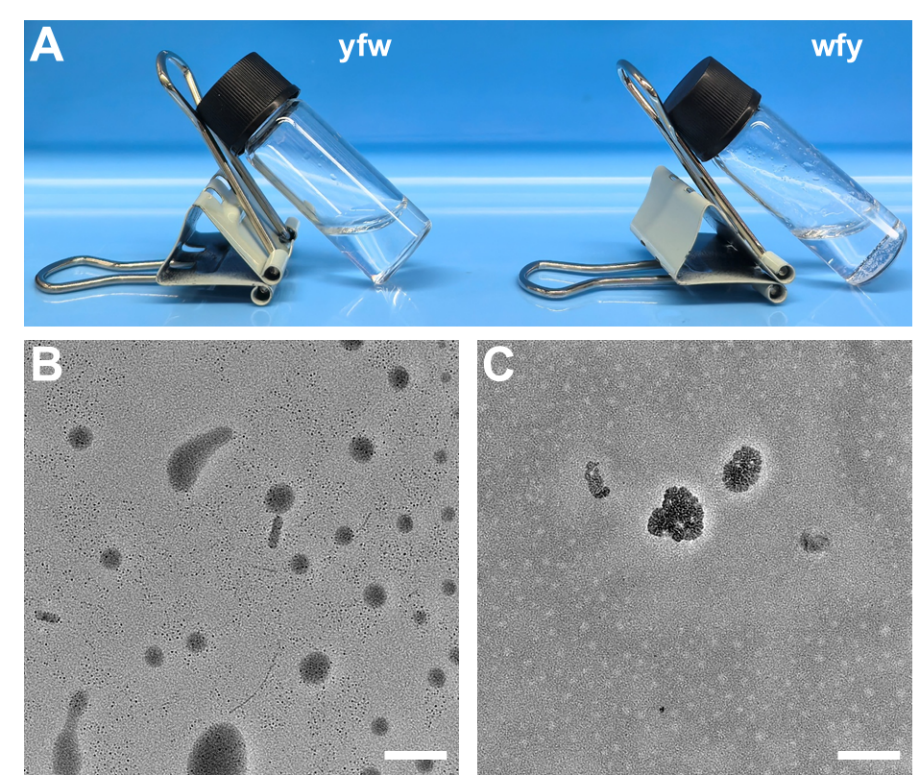
*

Figure S10. A) Optical photos and B, C) TEM images of nanostructures formed by B) yfw and C) wfy through “heating-cooling” strategy. The concentration of tripeptide is 3.0 mg/mL. Scale bar in B, C) is 100 nm.


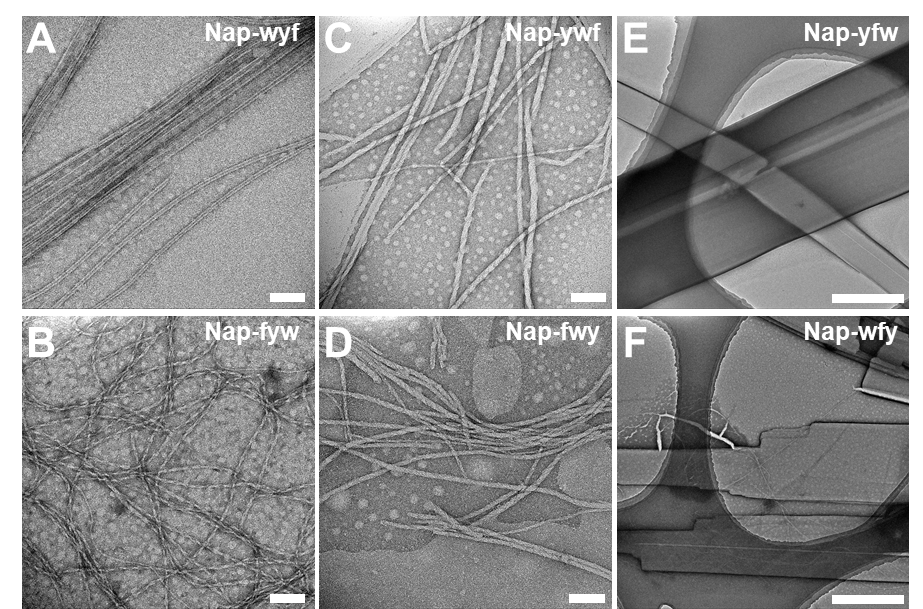


**Figure S11.** TEM images of nanostructures formed by tripeptides. The concentration of tripeptide is 3.0 mg/mL. Scale bar of A-D) and E, F) is 100 nm and 500 nm, respectively.


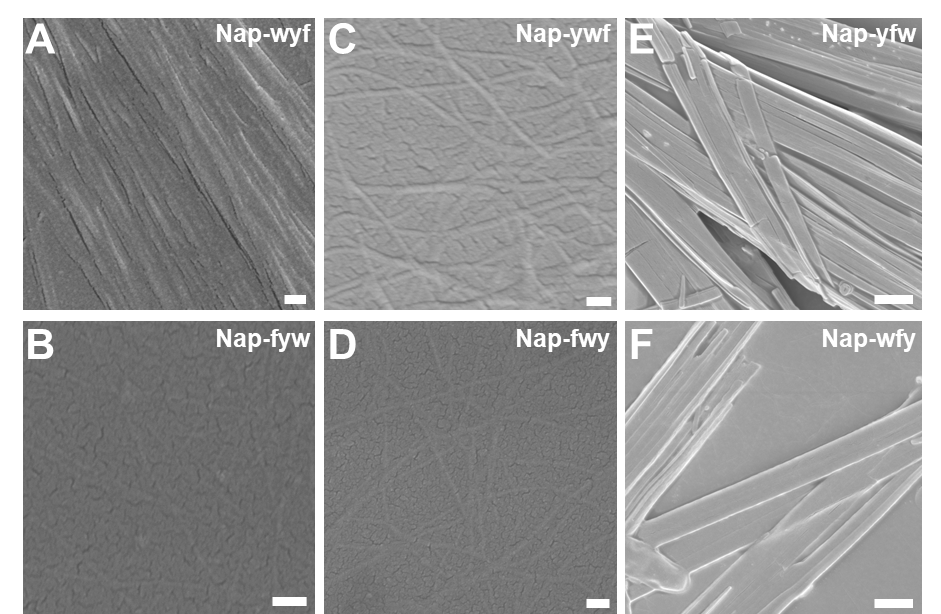


**Figure S12.** SEM images of nanostructures formed by tripeptides. The concentration of tripeptide is 3.0 mg/mL. Scale bar of A-D) and E, F) is 100 nm and 10 µm, respectively.


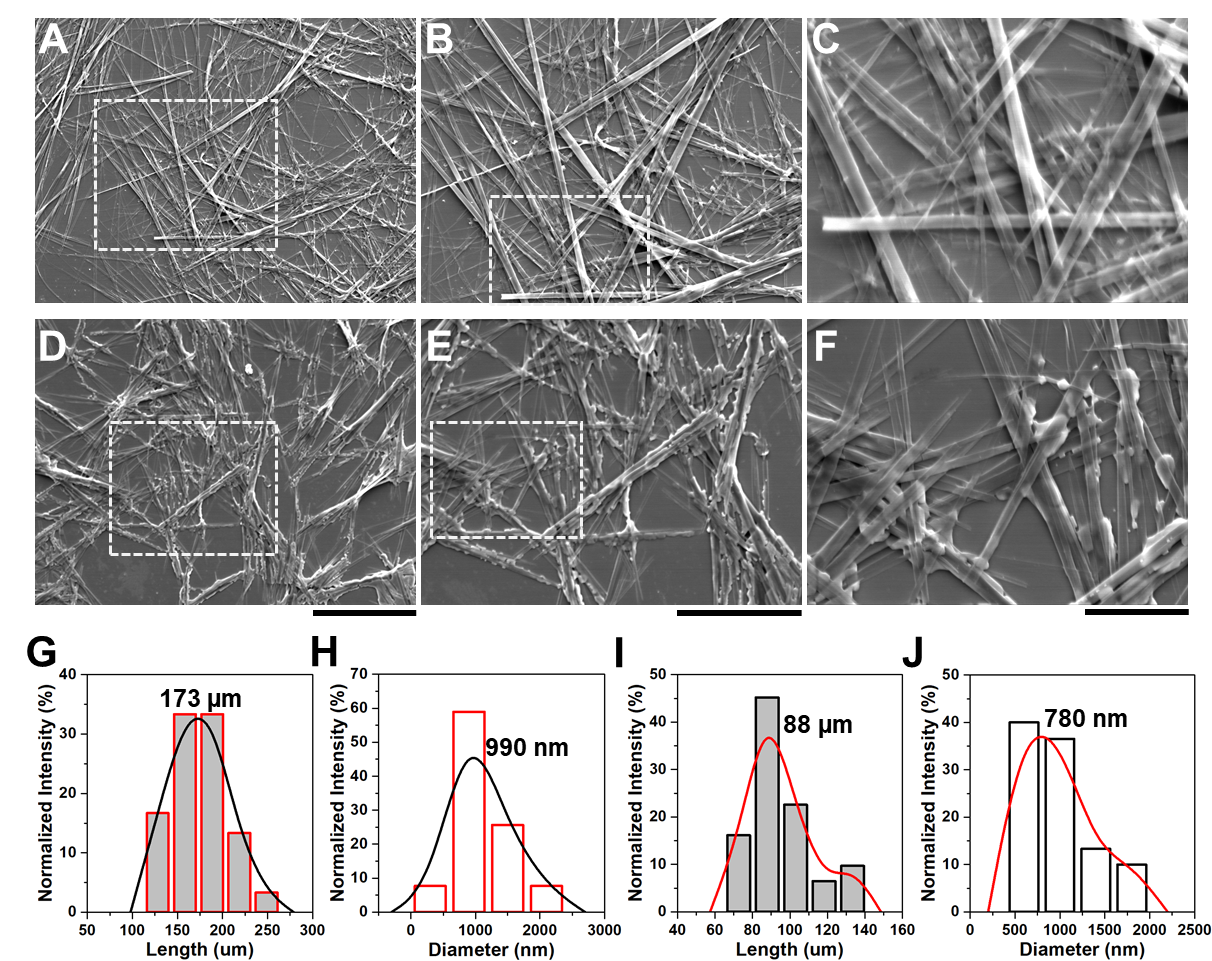


Figure S13. A-F) SEM images, statistic G, I) length and H, J) diameter of crystals formed by A-C, G, H) Nap-yfw and D-F, I, J) Nap-wfy, respectively. The concentration of Nap-yfw and Nap-wfy is 3.0 mg/mL. Scale bar of A, D), B, E), and C, F) is 50 µm, 30 µm, and 10 µm, respectively.


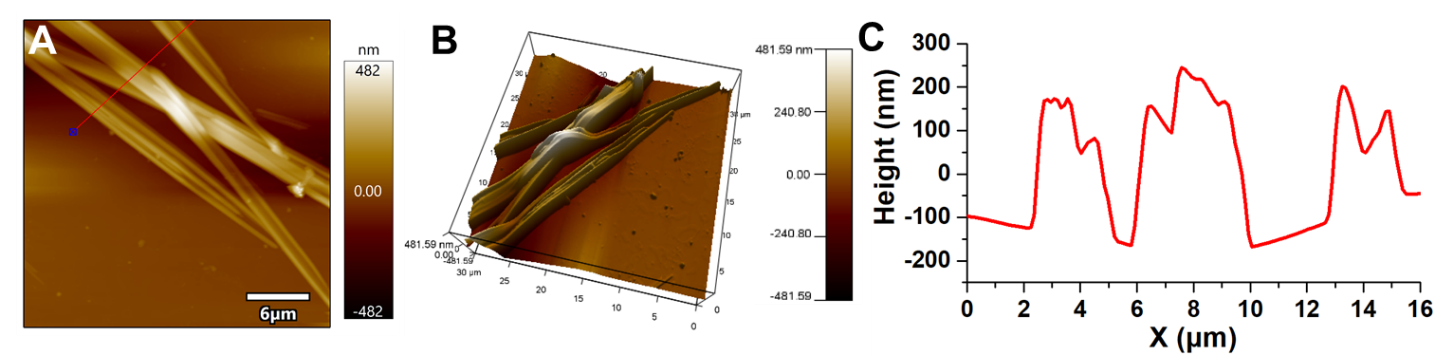


**Figure S14.** AFM images of crystals formed by **Nap-yfw**. The concentration of **Nap-yfw** is 3.0 mg/mL.


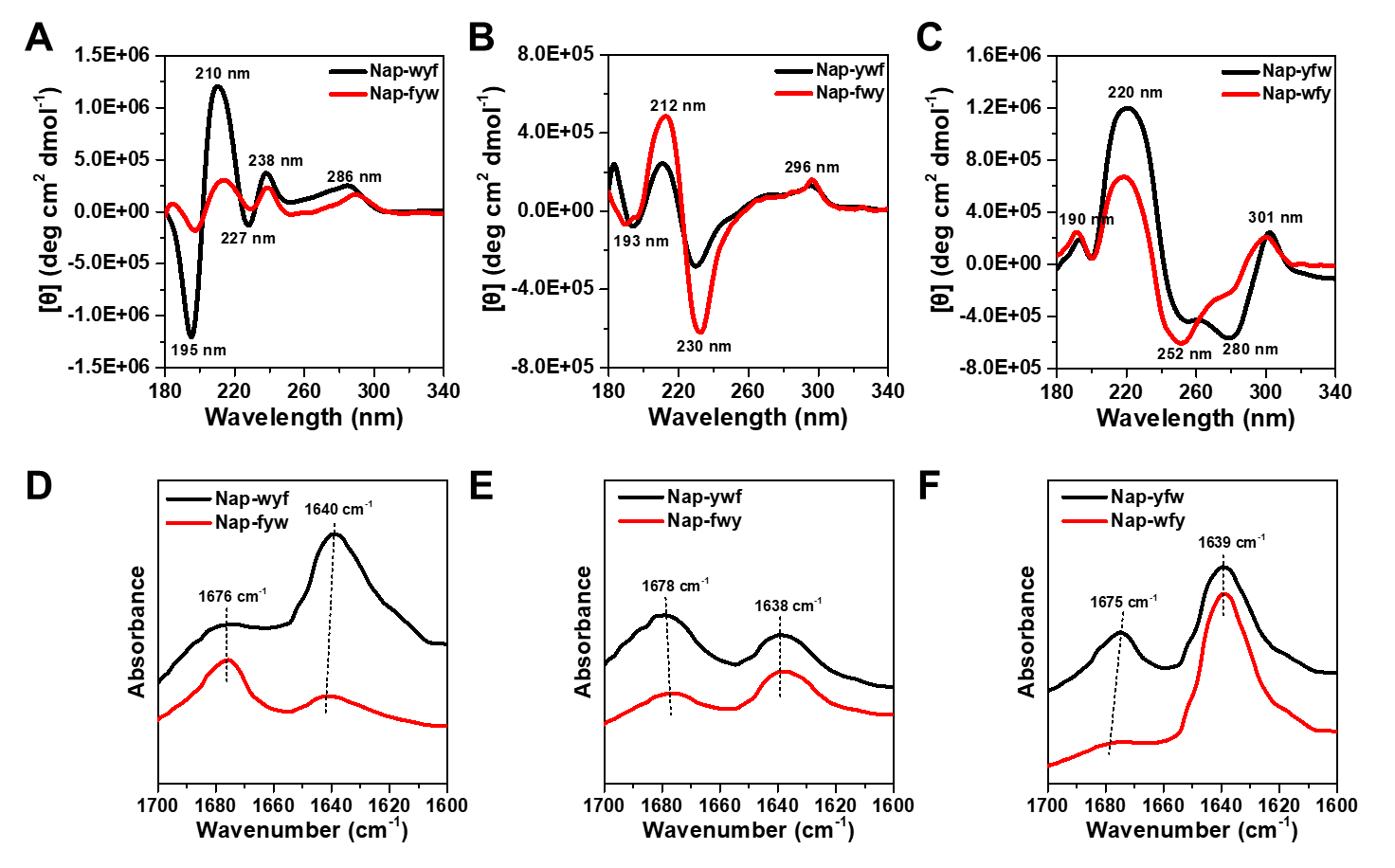


Figure S15. A-C) CD and D-F) FTIR spectra of nanostructures formed by tripeptides. The concentration of tripeptides is 3.0 mg/mL.


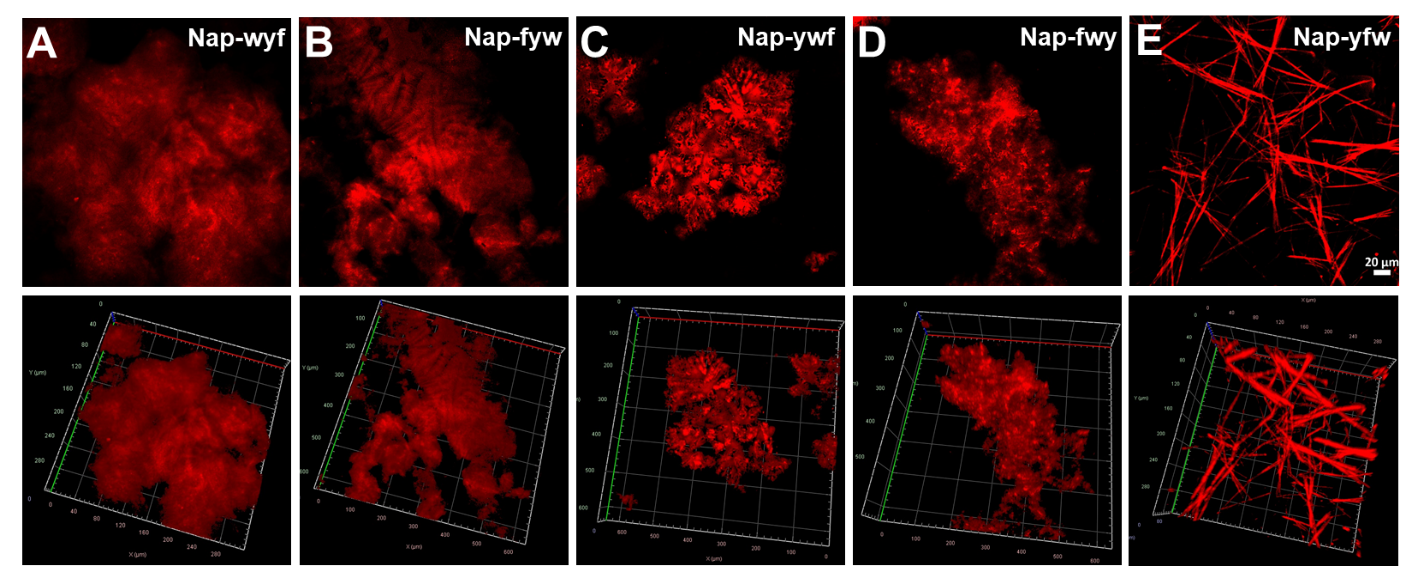


**Figure S16.** Congo red staining with A-D) hydrogels and E) crystals formed by tripeptides. The concentration of tripeptides and Congo red is 3.0 mg/mL and 1 mM, respectively.


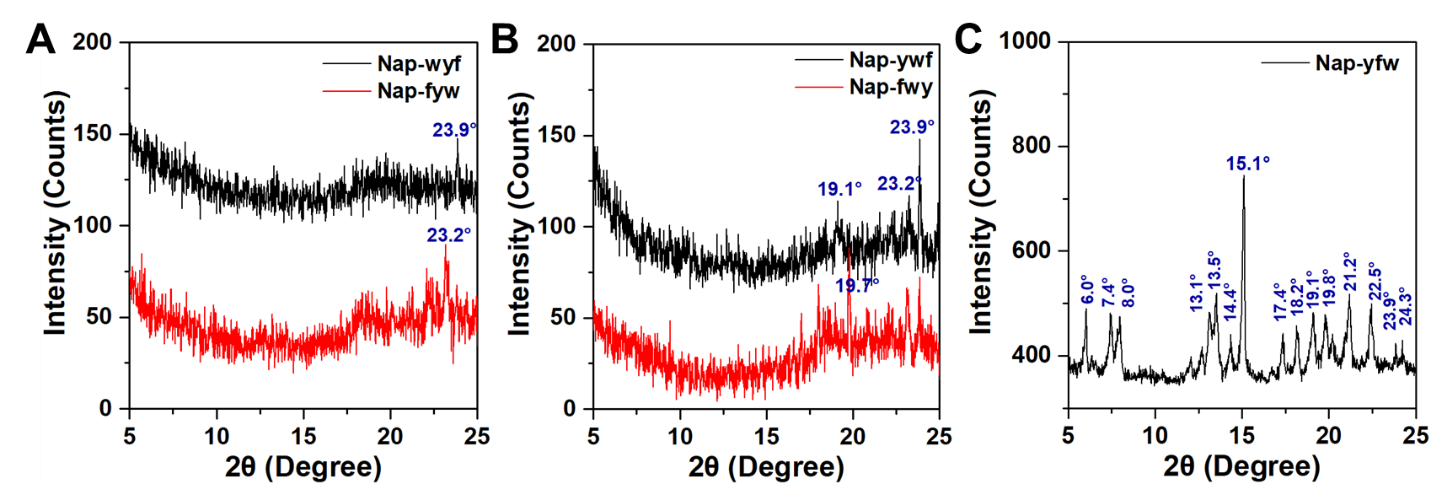


Figure S17. PXRD spectra of A, B) hydrogels and C) crystals formed by tripeptides. The concentration of tripeptides is 3.0 mg/mL.


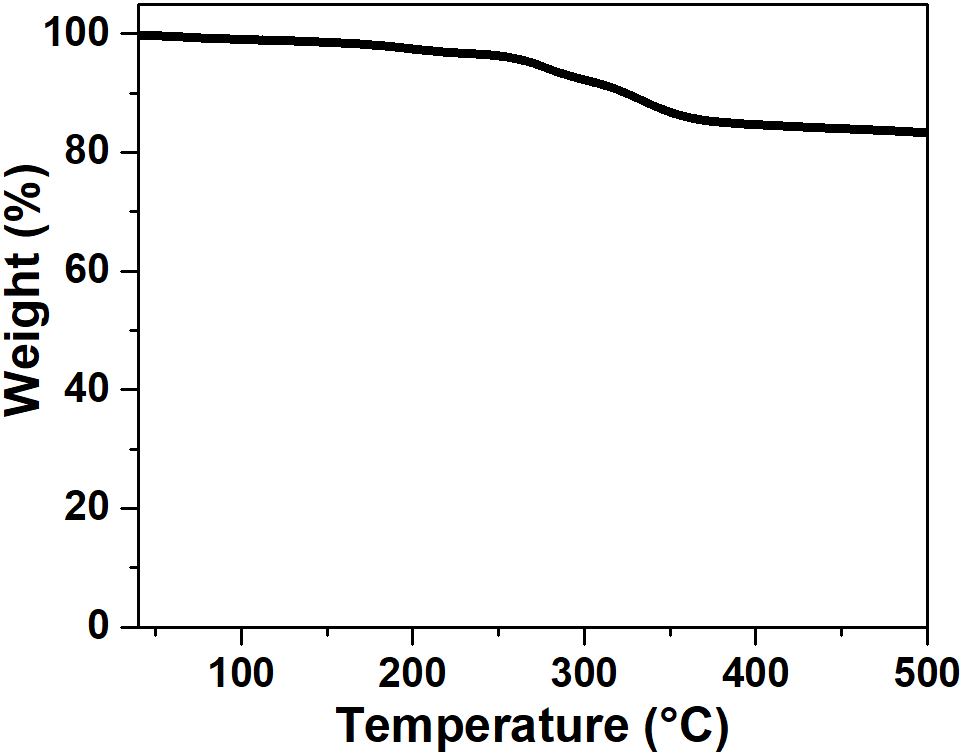


Figure S18. TGA analysis of Nap-yfw crystals.


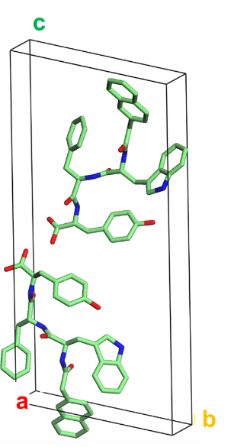


Figure S19. The unit cell of Nap-wfy crystals.


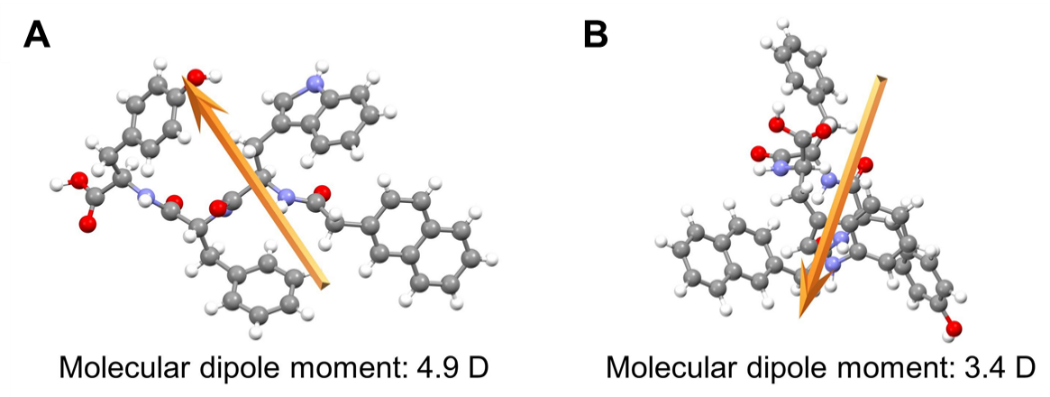


Figure S20. Molecule dipole moment of A) Nap-wfy and B) Nap-yfw.


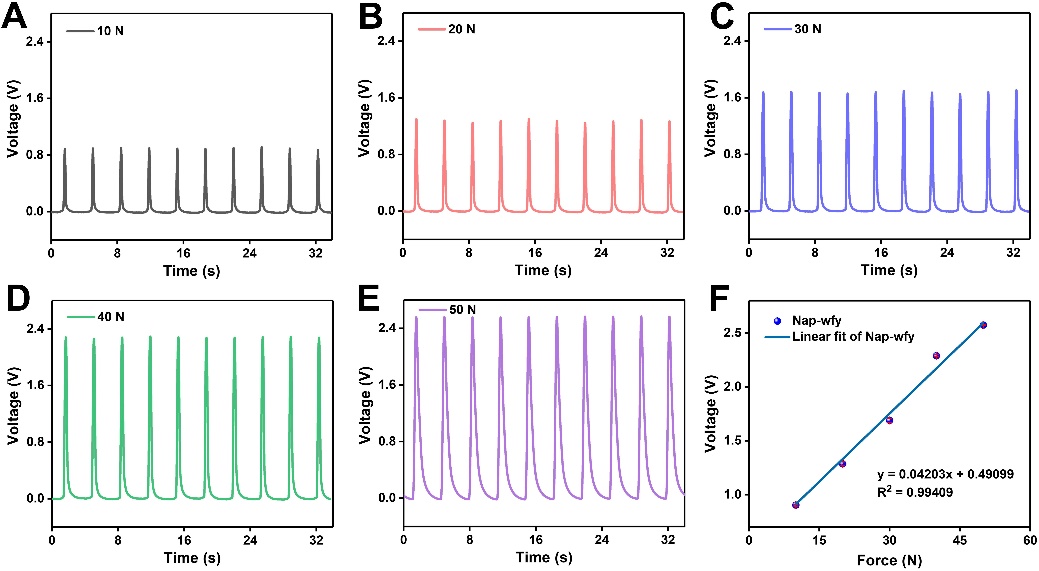


Figure S21. A-E) Forward open-circuit voltage output obtained from the piezoelectric energy harvester based on pure Nap-wfy crystals under various applied forces, A) 10 N, B) 20 N, C) 30 N, D) 40 N, and E) 50 N. F) Linear dependence of the voltage output on the applied force.


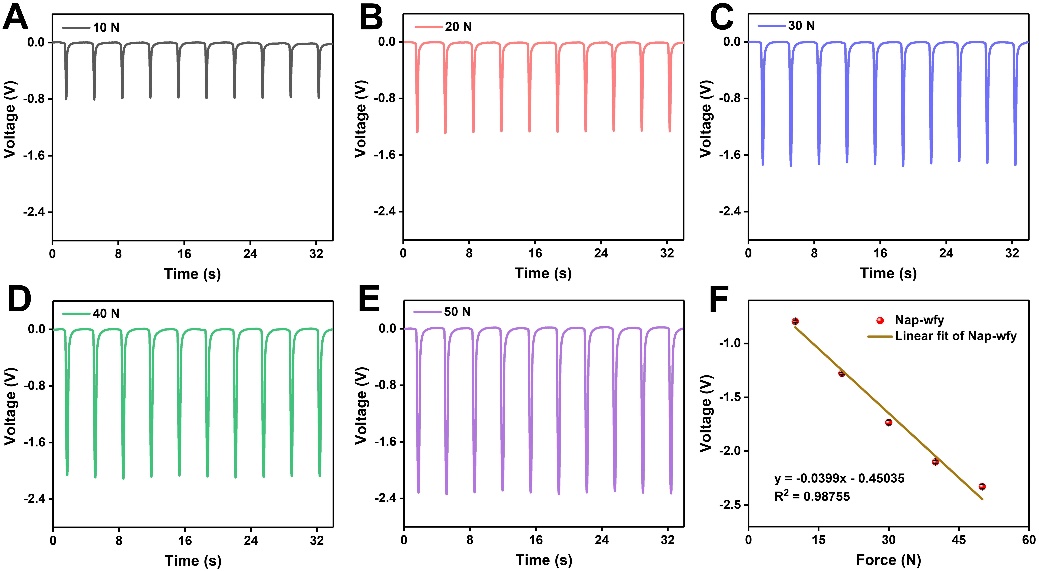


Figure S22. A-E) Reverse open-circuit voltage output obtained from the piezoelectric energy harvester based on pure Nap-wfy crystals under various applied forces, A) 10 N, B) 20 N, C) 30 N, D) 40 N, and E) 50 N. F) Linear dependence of the voltage output on the applied force.


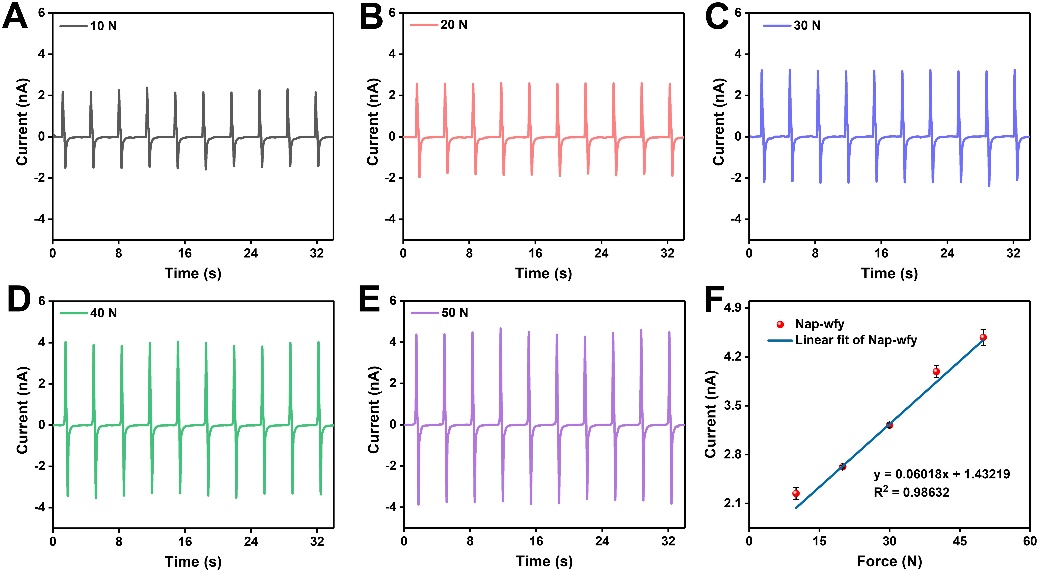


Figure S23. A-E) Forward short-circuit current output obtained from the piezoelectric energy harvester based on pure Nap-wfy crystals under various applied forces, A) 10 N, B) 20 N, C) 30 N, D) 40 N, and E) 50 N. F) Linear dependence of the current output on the applied force.


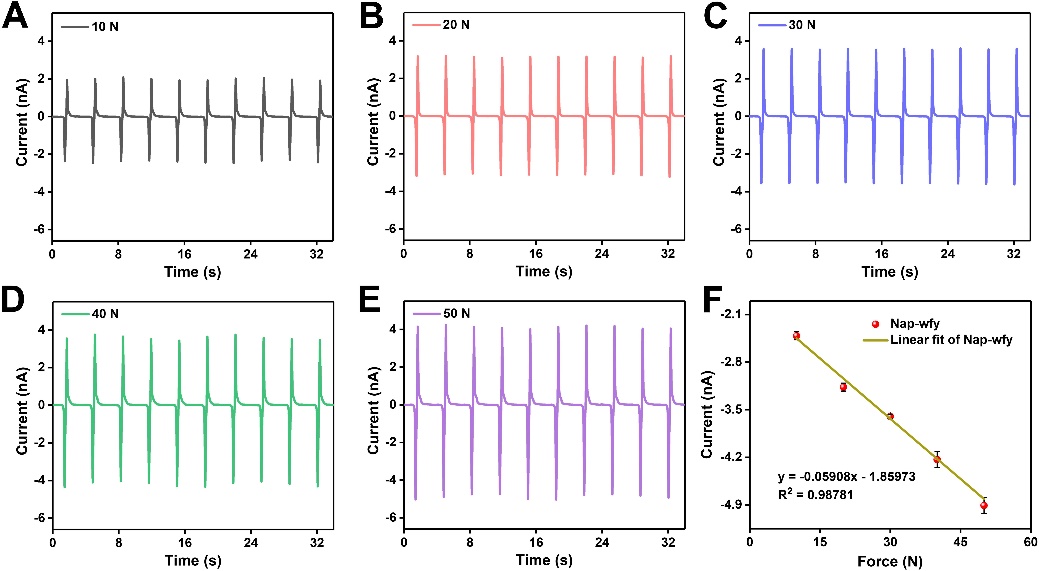


Figure S24. A-E) Reverse short-circuit current output obtained from the piezoelectric energy harvester based on pure Nap-wfy crystals under various applied forces, A) 10 N, B) 20 N, C) 30 N, D) 40 N, and E) 50 N. F) Linear dependence of the current output on the applied force.


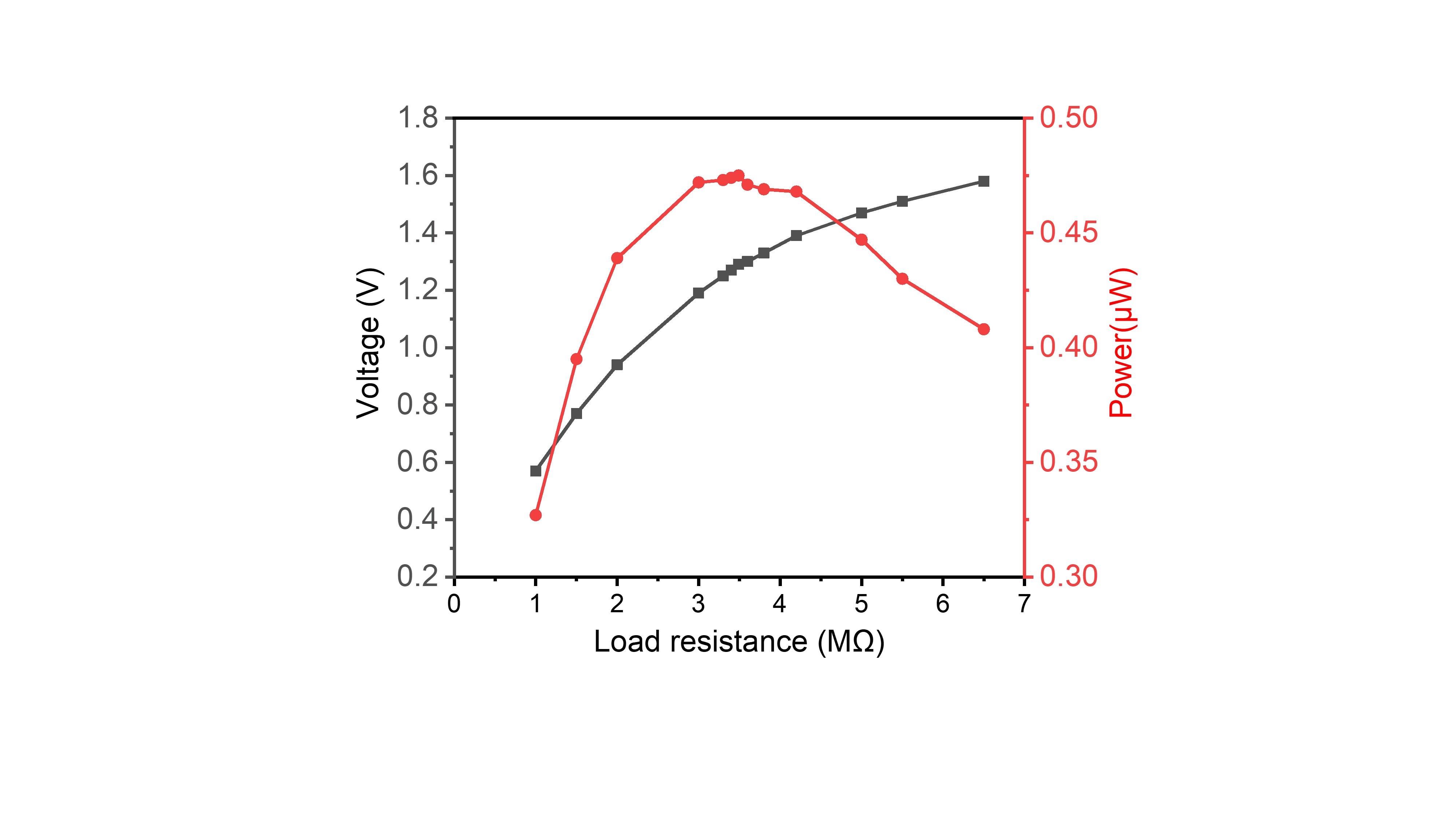


Figure S25. Dependence of the power output of the Nap-wfy crystal-based piezoelectric nanogenerators on the resistance of the external load under 50 N compressive force.


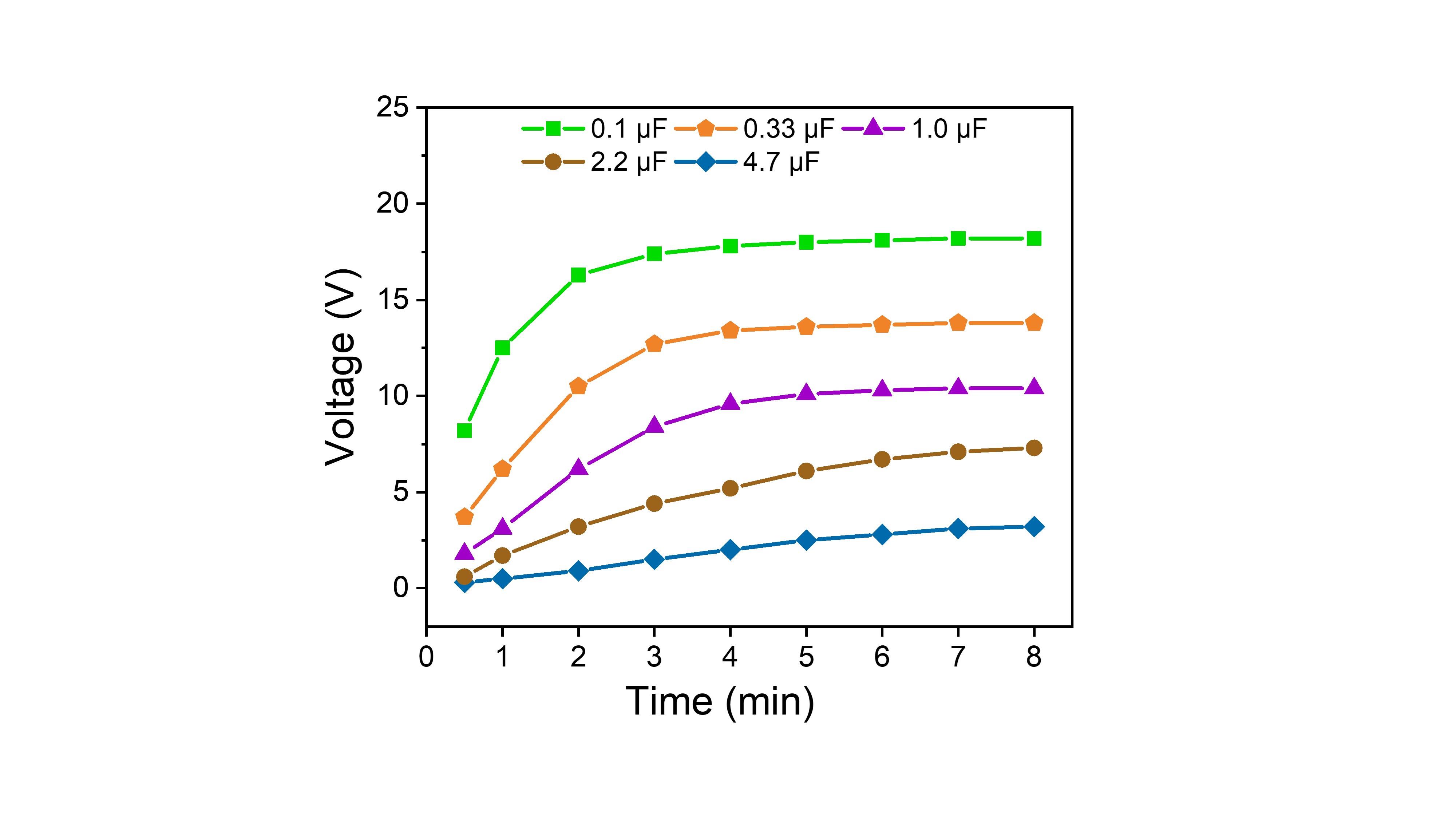


Figure S26. The charging capacity of capacitors of the Nap-wfy crystal-based piezoelectric nanogenerators under 50 N compressive force.


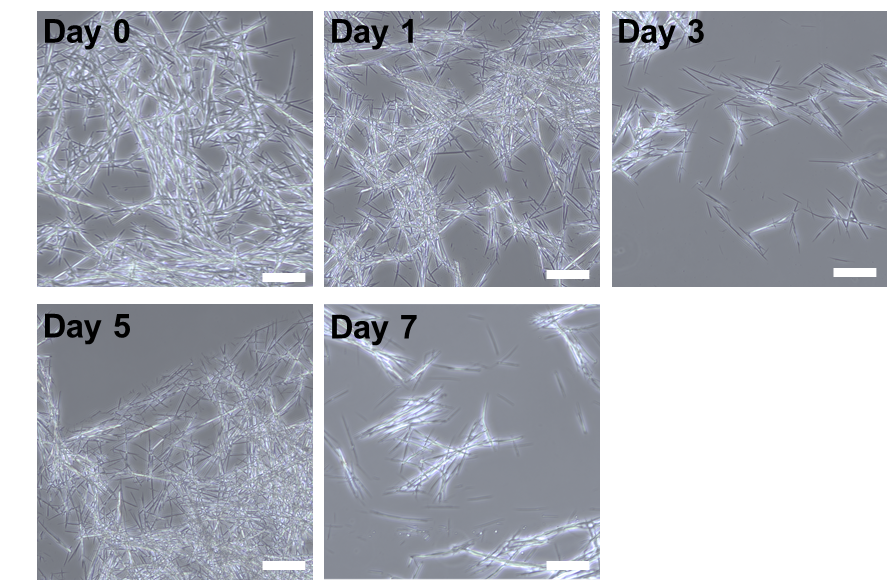


Figure S27. Optical microscope images of Nap-wfy crystals upon immersion in water for 7 days. Scale bar is 200 μm.

Figure S28. The voltage output of the Nap-wfy-based piezoelectric nanogenerators with 50 N force under relative humidity of around 90% for 1 day, 6 days and 12 days.

**S3. Supporting tables**

Table S1. Single-crystal structure of Nap-wfy crystals determined by MicroED.

| Molecule | Nap-wfy |
| --- | --- |
| Chemical formula | C_41_H_38_N_4_O_6_ |
| Molecular weight | 682.75 g·mol^-1^ |
| Temperature | 83(2) K |
| Wavelength | 0.02508 Å |
| Space group | Triclinic, P1 (No. 1) |
| Lattice parameters | *a* = 4.868(4) Å  *b* = 13.737(4) Å  *c* = 29.33(5) Å  *α* = 99.36(7)°  *β* = 90.24(12)°  *γ* = 90.20(5)° |
| Volume | 1935(4) Å^3^ |
| Z’ | 2 |
| Density | 1.172 g/cm^3^ |
| Resolution | 1.00 Å |
| Total diffraction points | 54459 |
| Independent diffraction points | 7187 |
| Integrity | 89.1 % |
| R_int_ | 0.2161 |
| Initial model generation method | ab initio |
| Hydrogen finishing | Geometric constraint |
| Goodness-of-fit on F^2^ | 1.442 |
| R_1_ [I ≥ 2sigma(I)] | 0.1715 |
| R_1_ [all data] | 0.2107 |
| wR_2_[I ≥ 2sigma(I)] | 0.3885 |
| wR_2_[all data] | 0.4040 |

Table S2. Atomic fractional coordinate x, y, z (Å) and equivalent isotropic displacement parameters U_eq_ (Å^2^) inside Nap-wfy single crystal.

| **Atom** | **x** | **y** | **z** | **U_eq_** |
| --- | --- | --- | --- | --- |
| O1 | 1.006(3) | 0.2342(12) | 1.0930(9) | 0.052(5) |
| N1 | 1.430(3) | 0.2268(9) | 1.1205(9) | 0.041(4) |
| H1 | 1.602976 | 0.213852 | 1.113568 | 0.049 |
| O2 | 1.709(3) | 0.1388(13) | 1.1907(9) | 0.059(8) |
| N2 | 1.278(3) | 0.0913(13) | 1.1984(11) | 0.070(7) |
| H2A | 1.102949 | 0.106290 | 1.196257 | 0.084 |
| O3 | 1.026(3) | -0.0009(16) | 1.2731(10) | 0.068(7) |
| N3 | 1.463(4) | 0.034(3) | 1.2958(10) | 0.126(11) |
| H3 | 1.630489 | 0.030577 | 1.284685 | 0.151 |
| O4 | 1.473(9) | -0.116(2) | 1.349(2) | 0.169(16) |
| H4B | 1.44(9) | -0.162(7) | 1.371(6) | 0.254 |
| O5 | 1.275(6) | -0.012(2) | 1.4052(13) | 0.146(12) |
| N5 | 0.999(3) | 0.5976(14) | 0.7317(11) | 0.061(5) |
| H5A | 0.827776 | 0.586378 | 0.738905 | 0.073 |
| N6 | 1.140(3) | 0.4117(14) | 0.6534(11) | 0.067(7) |
| H6B | 1.313706 | 0.430159 | 0.655979 | 0.080 |
| O7 | 0.931(19) | 0.097(3) | 0.496(3) | 0.64(16) |
| N7 | 0.979(5) | 0.287(3) | 0.5506(17) | 0.122(9) |
| H7 | 0.807751 | 0.300394 | 0.558839 | 0.147 |
| O8 | 1.421(3) | 0.6251(13) | 0.7585(9) | 0.055(6) |
| N8 | 1.060(4) | 0.5667(15) | 1.1804(14) | 0.091(7) |
| H8A | 0.938677 | 0.612824 | 1.189926 | 0.109 |
| O9 | 0.703(3) | 0.4601(13) | 0.6628(12) | 0.099(12) |
| N9 | 1.356(5) | 0.8881(15) | 0.6735(15) | 0.115(9) |
| H9 | 1.483327 | 0.927256 | 0.665059 | 0.138 |
| C9 | 0.865(2) | 0.3362(6) | 0.9649(5) | 0.064(6) |
| H9A | 0.800634 | 0.400642 | 0.962905 | 0.077 |
| C8 | 1.063(2) | 0.3229(7) | 0.9975(5) | 0.062(6) |
| H8 | 1.135269 | 0.378290 | 1.017738 | 0.074 |
| C7 | 1.157(2) | 0.2287(8) | 1.0005(5) | 0.053(4) |
| C6 | 1.051(2) | 0.1477(7) | 0.9709(6) | 0.063(6) |
| H6A | 1.115377 | 0.083261 | 0.972920 | 0.075 |
| C1 | 0.8527(17) | 0.1610(6) | 0.9383(5) | 0.068(6) |
| C10 | 0.7592(16) | 0.2552(6) | 0.9353(5) | 0.059(5) |
| C5 | 0.560(2) | 0.2685(8) | 0.9028(5) | 0.087(8) |
| H5 | 0.496559 | 0.332934 | 0.900737 | 0.105 |
| C4 | 0.455(2) | 0.1875(9) | 0.8732(6) | 0.073(7) |
| H4A | 0.319288 | 0.196597 | 0.850911 | 0.087 |
| C3 | 0.549(3) | 0.0933(8) | 0.8761(6) | 0.105(11) |
| H3A | 0.476651 | 0.037906 | 0.855917 | 0.126 |
| C2 | 0.747(2) | 0.0800(6) | 0.9087(6) | 0.082(8) |
| H2 | 0.811285 | 0.015550 | 0.910749 | 0.098 |
| O10 | 1.416(4) | 0.271(2) | 0.5740(14) | 0.114(10) |
| O11 | 1.186(6) | 0.148(3) | 0.4437(14) | 0.148(13) |
| H11C | 1.09(4) | 0.10(2) | 0.421(5) | 0.221 |
| C11 | 1.371(3) | 0.2171(13) | 1.0368(10) | 0.046(4) |
| H11A | 1.459776 | 0.151748 | 1.029137 | 0.055 |
| H11B | 1.515174 | 0.268421 | 1.037199 | 0.055 |
| O12 | 0.694(7) | 0.733(3) | 0.541(2) | 0.29(3) |
| H12A | 0.83(11) | 0.760(13) | 0.56(2) | 0.430 |
| C12 | 1.241(3) | 0.2265(11) | 1.0836(10) | 0.039(4) |
| O13 | 1.657(10) | 0.4943(19) | 1.3046(17) | 0.22(2) |
| H13A | 1.57(12) | 0.546(13) | 1.327(9) | 0.329 |
| C13 | 1.357(3) | 0.2468(13) | 1.1682(12) | 0.048(4) |
| H13C | 1.152308 | 0.249498 | 1.170641 | 0.057 |
| C14 | 1.459(3) | 0.1545(14) | 1.1859(11) | 0.053(6) |
| C15 | 1.332(3) | 0.0093(12) | 1.2137(10) | 0.054(4) |
| H15X | 1.534863 | -0.002137 | 1.211218 | 0.065 |
| C16 | 1.251(4) | 0.0119(17) | 1.2635(11) | 0.071(5) |
| C17 | 1.439(4) | 0.0607(18) | 1.3447(12) | 0.096(7) |
| H17 | 1.259488 | 0.096342 | 1.349096 | 0.116 |
| C18 | 1.398(5) | -0.029(2) | 1.3678(12) | 0.138(11) |
| C19 | 1.475(4) | 0.3398(13) | 1.1945(13) | 0.069(6) |
| H19A | 1.671879 | 0.344499 | 1.186654 | 0.083 |
| H19B | 1.462430 | 0.337987 | 1.228024 | 0.083 |
| C20 | 1.326(5) | 0.4300(17) | 1.1836(14) | 0.084(7) |
| C21 | 1.390(3) | 0.4787(11) | 1.1399(7) | 0.083(6) |
| C22 | 1.579(2) | 0.4529(9) | 1.1046(7) | 0.085(7) |
| H22 | 1.697779 | 0.398317 | 1.105155 | 0.102 |
| C23 | 1.594(3) | 0.5068(11) | 1.0685(7) | 0.088(7) |
| H23 | 1.723136 | 0.489130 | 1.044347 | 0.106 |
| C24 | 1.421(3) | 0.5866(12) | 1.0676(7) | 0.106(8) |
| H24 | 1.430879 | 0.623501 | 1.042958 | 0.127 |
| C25 | 1.232(3) | 0.6125(9) | 1.1029(8) | 0.104(8) |
| H25 | 1.113263 | 0.667061 | 1.102379 | 0.125 |
| C26 | 1.217(3) | 0.5586(10) | 1.1391(7) | 0.105(8) |
| C28 | 1.183(3) | -0.0807(12) | 1.1835(11) | 0.056(5) |
| H28A | 1.242891 | -0.142804 | 1.193372 | 0.067 |
| H28B | 0.981786 | -0.074624 | 1.188153 | 0.067 |
| C29 | 1.247(2) | -0.0837(9) | 1.1333(6) | 0.060(5) |
| C30 | 1.097(2) | -0.0275(9) | 1.1067(7) | 0.077(6) |
| H30 | 0.948909 | 0.011719 | 1.120051 | 0.092 |
| C31 | 1.165(3) | -0.0287(11) | 1.0606(7) | 0.093(7) |
| H31 | 1.062329 | 0.009693 | 1.042442 | 0.111 |
| C32 | 1.382(3) | -0.0861(11) | 1.0411(6) | 0.088(7) |
| H32 | 1.428056 | -0.086968 | 1.009611 | 0.106 |
| C33 | 1.532(2) | -0.1424(9) | 1.0677(7) | 0.083(7) |
| H33 | 1.680365 | -0.181604 | 1.054387 | 0.100 |
| C34 | 1.465(2) | -0.1412(8) | 1.1138(7) | 0.076(6) |
| H34 | 1.566950 | -0.179580 | 1.131996 | 0.091 |
| C35 | 1.630(5) | 0.128(2) | 1.3672(14) | 0.118(9) |
| H35A | 1.812603 | 0.096118 | 1.363800 | 0.142 |
| H35B | 1.585640 | 0.138785 | 1.400590 | 0.142 |
| C38 | 1.673(5) | 0.4086(15) | 1.3229(12) | 0.182(18) |
| C39 | 1.487(5) | 0.3924(15) | 1.3568(12) | 0.20(2) |
| H39 | 1.367281 | 0.443673 | 1.369893 | 0.241 |
| C40 | 1.478(4) | 0.3013(17) | 1.3715(10) | 0.167(18) |
| H40 | 1.350827 | 0.290196 | 1.394649 | 0.200 |
| C36 | 1.654(4) | 0.2263(13) | 1.3523(9) | 0.105(9) |
| C37 | 1.839(4) | 0.2425(15) | 1.3184(9) | 0.138(13) |
| H37 | 1.959453 | 0.191257 | 1.305307 | 0.166 |
| C42 | 1.849(4) | 0.3337(18) | 1.3037(9) | 0.141(13) |
| H42 | 1.975911 | 0.344734 | 1.280551 | 0.170 |
| C41 | 1.139(5) | 0.4812(19) | 1.2051(16) | 0.100(8) |
| H41 | 1.057518 | 0.468373 | 1.232955 | 0.120 |
| C49 | 1.543(2) | 0.8107(6) | 0.8870(5) | 0.073(8) |
| H49 | 1.604003 | 0.877205 | 0.890160 | 0.088 |
| C50 | 1.342(2) | 0.7761(7) | 0.8545(5) | 0.068(8) |
| H50 | 1.265779 | 0.818910 | 0.835466 | 0.082 |
| C51 | 1.252(2) | 0.6788(7) | 0.8500(5) | 0.053(5) |
| C52 | 1.363(2) | 0.6161(6) | 0.8779(5) | 0.061(6) |
| H52 | 1.301847 | 0.549643 | 0.874730 | 0.073 |
| C43 | 1.5641(18) | 0.6508(5) | 0.9104(5) | 0.059(6) |
| C48 | 1.6538(16) | 0.7481(5) | 0.9149(4) | 0.053(6) |
| C47 | 1.855(2) | 0.7827(6) | 0.9474(5) | 0.063(7) |
| H47 | 1.916079 | 0.849175 | 0.950554 | 0.075 |
| C46 | 1.966(2) | 0.7200(8) | 0.9753(5) | 0.061(7) |
| H46 | 2.103232 | 0.743692 | 0.997534 | 0.074 |
| C45 | 1.876(3) | 0.6227(8) | 0.9707(6) | 0.090(10) |
| H45 | 1.952163 | 0.579911 | 0.989820 | 0.108 |
| C44 | 1.675(3) | 0.5881(7) | 0.9383(6) | 0.129(14) |
| H44 | 1.613941 | 0.521612 | 0.935128 | 0.155 |
| C53 | 1.045(3) | 0.6370(12) | 0.8108(11) | 0.048(4) |
| H53A | 0.977152 | 0.571904 | 0.816369 | 0.058 |
| H53B | 0.885634 | 0.681779 | 0.811982 | 0.058 |
| C54 | 1.162(3) | 0.6265(12) | 0.7671(10) | 0.044(4) |
| C55 | 1.055(3) | 0.5817(15) | 0.6839(12) | 0.055(4) |
| H55 | 1.259302 | 0.583760 | 0.680873 | 0.066 |
| C56 | 0.965(3) | 0.4729(17) | 0.6653(12) | 0.076(8) |
| C57 | 1.080(3) | 0.3139(16) | 0.6359(11) | 0.077(5) |
| H57 | 0.877844 | 0.303461 | 0.639200 | 0.092 |
| C58 | 1.155(4) | 0.2824(19) | 0.5853(13) | 0.088(6) |
| C59 | 1.027(4) | 0.2729(18) | 0.5038(13) | 0.111(7) |
| H66 | 1.208830 | 0.303992 | 0.499099 | 0.133 |
| C60 | 1.043(5) | 0.164(2) | 0.4815(13) | 0.120(10) |
| C61 | 0.941(4) | 0.6568(17) | 0.6560(13) | 0.075(6) |
| H61A | 0.969879 | 0.632805 | 0.622652 | 0.090 |
| H61B | 0.741136 | 0.663986 | 0.661332 | 0.090 |
| C62 | 1.079(4) | 0.7556(19) | 0.6693(15) | 0.093(7) |
| C73 | 0.829(2) | 0.8276(8) | 0.7451(7) | 0.069(6) |
| H73 | 0.708102 | 0.773029 | 0.744832 | 0.083 |
| C74 | 0.816(3) | 0.9073(10) | 0.7810(7) | 0.093(7) |
| H74 | 0.686990 | 0.907103 | 0.805155 | 0.112 |
| C75 | 0.993(3) | 0.9872(9) | 0.7814(7) | 0.093(7) |
| H75 | 0.984132 | 1.041633 | 0.805938 | 0.112 |
| C76 | 1.182(3) | 0.9875(8) | 0.7461(8) | 0.093(7) |
| H76 | 1.302388 | 1.042090 | 0.746398 | 0.111 |
| C64 | 1.194(3) | 0.9078(10) | 0.7102(7) | 0.097(7) |
| C63 | 1.018(3) | 0.8279(9) | 0.7098(6) | 0.073(6) |
| C65 | 1.280(4) | 0.7839(15) | 0.6484(13) | 0.090(7) |
| H65 | 1.369058 | 0.748147 | 0.622129 | 0.108 |
| C66 | 1.233(4) | 0.2477(18) | 0.6652(13) | 0.088(6) |
| H66A | 1.433192 | 0.256477 | 0.662087 | 0.105 |
| H66B | 1.187282 | 0.177657 | 0.653638 | 0.105 |
| C70 | 1.049(3) | 0.3347(12) | 0.8104(7) | 0.103(8) |
| H70 | 1.014452 | 0.355525 | 0.842295 | 0.124 |
| C71 | 0.886(3) | 0.2619(12) | 0.7849(8) | 0.110(9) |
| H71 | 0.739978 | 0.233038 | 0.799489 | 0.132 |
| C72 | 0.937(3) | 0.2315(10) | 0.7382(8) | 0.100(8) |
| H72 | 0.825620 | 0.181764 | 0.720808 | 0.119 |
| C67 | 1.151(3) | 0.2738(11) | 0.7169(7) | 0.083(6) |
| C68 | 1.314(2) | 0.3466(11) | 0.7423(8) | 0.094(8) |
| H68 | 1.460212 | 0.375464 | 0.727737 | 0.113 |
| C69 | 1.263(3) | 0.3770(10) | 0.7890(8) | 0.103(8) |
| H69 | 1.374574 | 0.426739 | 0.806418 | 0.124 |
| C77 | 0.784(4) | 0.4317(18) | 0.4968(12) | 0.157(15) |
| C78 | 0.918(7) | 0.498(2) | 0.4737(16) | 0.89(19) |
| H78 | 1.026123 | 0.475232 | 0.447244 | 1.069 |
| C81 | 0.895(9) | 0.599(2) | 0.4891(19) | 0.38(4) |
| H81 | 0.986466 | 0.644840 | 0.473313 | 0.459 |
| C85 | 0.737(6) | 0.6332(17) | 0.5278(18) | 0.29(3) |
| C82 | 0.602(5) | 0.567(2) | 0.5509(13) | 0.182(17) |
| H82 | 0.494055 | 0.589764 | 0.577349 | 0.219 |
| C79 | 0.626(5) | 0.466(2) | 0.5355(11) | 0.204(19) |
| H79 | 0.533709 | 0.420155 | 0.551281 | 0.245 |
| C80 | 0.807(3) | 0.328(2) | 0.4798(13) | 0.118(9) |
| H80A | 0.849407 | 0.318795 | 0.446368 | 0.142 |
| H80B | 0.627155 | 0.296069 | 0.483142 | 0.142 |
| H33 | 1.680365 | -0.181604 | 1.054387 | 0.100 |
| C34 | 1.465(2) | -0.1412(8) | 1.1138(7) | 0.076(6) |
| H34 | 1.566950 | -0.179580 | 1.131996 | 0.091 |
| C35 | 1.630(5) | 0.128(2) | 1.3672(14) | 0.118(9) |
| H35A | 1.812603 | 0.096118 | 1.363800 | 0.142 |
| H35B | 1.585640 | 0.138785 | 1.400590 | 0.142 |
| C38 | 1.673(5) | 0.4086(15) | 1.3229(12) | 0.182(18) |
| C39 | 1.487(5) | 0.3924(15) | 1.3568(12) | 0.20(2) |
| H39 | 1.367281 | 0.443673 | 1.369893 | 0.241 |
| C40 | 1.478(4) | 0.3013(17) | 1.3715(10) | 0.167(18) |
| H40 | 1.350827 | 0.290196 | 1.394649 | 0.200 |
| C36 | 1.654(4) | 0.2263(13) | 1.3523(9) | 0.105(9) |
| C37 | 1.839(4) | 0.2425(15) | 1.3184(9) | 0.138(13) |
| H37 | 1.959453 | 0.191257 | 1.305307 | 0.166 |
| C42 | 1.849(4) | 0.3337(18) | 1.3037(9) | 0.141(13) |
| H42 | 1.975911 | 0.344734 | 1.280551 | 0.170 |
| C41 | 1.139(5) | 0.4812(19) | 1.2051(16) | 0.100(8) |
| H41 | 1.057518 | 0.468373 | 1.232955 | 0.120 |
| C49 | 1.543(2) | 0.8107(6) | 0.8870(5) | 0.073(8) |
| H49 | 1.604003 | 0.877205 | 0.890160 | 0.088 |
| C50 | 1.342(2) | 0.7761(7) | 0.8545(5) | 0.068(8) |
| H50 | 1.265779 | 0.818910 | 0.835466 | 0.082 |
| C51 | 1.252(2) | 0.6788(7) | 0.8500(5) | 0.053(5) |
| C52 | 1.363(2) | 0.6161(6) | 0.8779(5) | 0.061(6) |
| H52 | 1.301847 | 0.549643 | 0.874730 | 0.073 |
| C43 | 1.5641(18) | 0.6508(5) | 0.9104(5) | 0.059(6) |
| C48 | 1.6538(16) | 0.7481(5) | 0.9149(4) | 0.053(6) |
| C47 | 1.855(2) | 0.7827(6) | 0.9474(5) | 0.063(7) |
| H47 | 1.916079 | 0.849175 | 0.950554 | 0.075 |
| C46 | 1.966(2) | 0.7200(8) | 0.9753(5) | 0.061(7) |
| H46 | 2.103232 | 0.743692 | 0.997534 | 0.074 |
| C45 | 1.876(3) | 0.6227(8) | 0.9707(6) | 0.090(10) |
| H45 | 1.952163 | 0.579911 | 0.989820 | 0.108 |
| C44 | 1.675(3) | 0.5881(7) | 0.9383(6) | 0.129(14) |
| H44 | 1.613941 | 0.521612 | 0.935128 | 0.155 |
| C53 | 1.045(3) | 0.6370(12) | 0.8108(11) | 0.048(4) |
| H53A | 0.977152 | 0.571904 | 0.816369 | 0.058 |
| H53B | 0.885634 | 0.681779 | 0.811982 | 0.058 |
| C54 | 1.162(3) | 0.6265(12) | 0.7671(10) | 0.044(4) |
| C55 | 1.055(3) | 0.5817(15) | 0.6839(12) | 0.055(4) |
| H55 | 1.259302 | 0.583760 | 0.680873 | 0.066 |
| C56 | 0.965(3) | 0.4729(17) | 0.6653(12) | 0.076(8) |
| C57 | 1.080(3) | 0.3139(16) | 0.6359(11) | 0.077(5) |
| H57 | 0.877844 | 0.303461 | 0.639200 | 0.092 |
| C58 | 1.155(4) | 0.2824(19) | 0.5853(13) | 0.088(6) |
| C59 | 1.027(4) | 0.2729(18) | 0.5038(13) | 0.111(7) |
| H66 | 1.208830 | 0.303992 | 0.499099 | 0.133 |
| C60 | 1.043(5) | 0.164(2) | 0.4815(13) | 0.120(10) |
| C61 | 0.941(4) | 0.6568(17) | 0.6560(13) | 0.075(6) |
| H61A | 0.969879 | 0.632805 | 0.622652 | 0.090 |
| H61B | 0.741136 | 0.663986 | 0.661332 | 0.090 |
| C62 | 1.079(4) | 0.7556(19) | 0.6693(15) | 0.093(7) |
| C73 | 0.829(2) | 0.8276(8) | 0.7451(7) | 0.069(6) |
| H73 | 0.708102 | 0.773029 | 0.744832 | 0.083 |
| C74 | 0.816(3) | 0.9073(10) | 0.7810(7) | 0.093(7) |
| H74 | 0.686990 | 0.907103 | 0.805155 | 0.112 |
| C75 | 0.993(3) | 0.9872(9) | 0.7814(7) | 0.093(7) |
| H75 | 0.984132 | 1.041633 | 0.805938 | 0.112 |
| C76 | 1.182(3) | 0.9875(8) | 0.7461(8) | 0.093(7) |
| H76 | 1.302388 | 1.042090 | 0.746398 | 0.111 |
| C64 | 1.194(3) | 0.9078(10) | 0.7102(7) | 0.097(7) |
| C63 | 1.018(3) | 0.8279(9) | 0.7098(6) | 0.073(6) |
| C65 | 1.280(4) | 0.7839(15) | 0.6484(13) | 0.090(7) |
| H65 | 1.369058 | 0.748147 | 0.622129 | 0.108 |
| C66 | 1.233(4) | 0.2477(18) | 0.6652(13) | 0.088(6) |
| H66A | 1.433192 | 0.256477 | 0.662087 | 0.105 |
| H66B | 1.187282 | 0.177657 | 0.653638 | 0.105 |
| C70 | 1.049(3) | 0.3347(12) | 0.8104(7) | 0.103(8) |
| H70 | 1.014452 | 0.355525 | 0.842295 | 0.124 |
| C71 | 0.886(3) | 0.2619(12) | 0.7849(8) | 0.110(9) |
| H71 | 0.739978 | 0.233038 | 0.799489 | 0.132 |
| C72 | 0.937(3) | 0.2315(10) | 0.7382(8) | 0.100(8) |
| H72 | 0.825620 | 0.181764 | 0.720808 | 0.119 |
| C67 | 1.151(3) | 0.2738(11) | 0.7169(7) | 0.083(6) |
| C68 | 1.314(2) | 0.3466(11) | 0.7423(8) | 0.094(8) |
| H68 | 1.460212 | 0.375464 | 0.727737 | 0.113 |
| C69 | 1.263(3) | 0.3770(10) | 0.7890(8) | 0.103(8) |
| H69 | 1.374574 | 0.426739 | 0.806418 | 0.124 |
| C77 | 0.784(4) | 0.4317(18) | 0.4968(12) | 0.157(15) |
| C78 | 0.918(7) | 0.498(2) | 0.4737(16) | 0.89(19) |
| H78 | 1.026123 | 0.475232 | 0.447244 | 1.069 |
| C81 | 0.895(9) | 0.599(2) | 0.4891(19) | 0.38(4) |
| H81 | 0.986466 | 0.644840 | 0.473313 | 0.459 |
| C85 | 0.737(6) | 0.6332(17) | 0.5278(18) | 0.29(3) |
| C82 | 0.602(5) | 0.567(2) | 0.5509(13) | 0.182(17) |
| H82 | 0.494055 | 0.589764 | 0.577349 | 0.219 |
| C79 | 0.626(5) | 0.466(2) | 0.5355(11) | 0.204(19) |
| H79 | 0.533709 | 0.420155 | 0.551281 | 0.245 |
| C80 | 0.807(3) | 0.328(2) | 0.4798(13) | 0.118(9) |
| H80A | 0.849407 | 0.318795 | 0.446368 | 0.142 |
| H80B | 0.627155 | 0.296069 | 0.483142 | 0.142 |

Table S3. Atomic anisotropic displacement parameters (Å^2^) inside Nap-wfy single crystal.

| **Atom** | **U_11_** | **U_22_** | **U_33_** | **U_23_** | **U_13_** | **U_12_** |
| --- | --- | --- | --- | --- | --- | --- |
| O1 | 0.014(5) | 0.080(10) | 0.059(17) | 0.002(11) | -0.002(6) | -0.011(6) |
| N1 | 0.029(7) | 0.016(6) | 0.080(10) | 0.013(8) | -0.006(7) | 0.004(5) |
| O2 | 0.009(4) | 0.084(11) | 0.10(2) | 0.056(13) | 0.005(7) | -0.007(5) |
| N2 | 0.006(6) | 0.065(7) | 0.15(2) | 0.045(11) | 0.028(9) | 0.008(5) |
| O3 | 0.012(6) | 0.122(14) | 0.065(18) | -0.003(14) | -0.001(7) | -0.009(7) |
| N3 | 0.001(7) | 0.26(3) | 0.111(14) | 0.015(17) | 0.002(9) | 0.001(11) |
| O4 | 0.15(3) | 0.172(17) | 0.19(5) | 0.06(3) | 0.02(3) | 0.01(2) |
| O5 | 0.11(2) | 0.136(19) | 0.21(3) | 0.07(2) | 0.04(2) | -0.024(16) |
| N5 | 0.013(7) | 0.091(11) | 0.081(11) | 0.021(9) | 0.010(7) | -0.027(7) |
| N6 | 0.004(6) | 0.080(7) | 0.12(2) | 0.023(9) | 0.019(9) | 0.000(5) |
| O7 | 1.1(3) | 0.21(3) | 0.6(2) | -0.05(6) | 0.7(2) | -0.14(8) |
| N7 | 0.018(9) | 0.21(3) | 0.137(15) | 0.022(18) | 0.009(10) | -0.009(12) |
| O8 | 0.006(5) | 0.087(11) | 0.076(19) | 0.031(12) | -0.002(6) | -0.002(5) |
| N8 | 0.057(11) | 0.058(10) | 0.15(2) | 0.010(12) | -0.012(11) | -0.011(7) |
| O9 | 0.001(5) | 0.068(10) | 0.22(4) | 0.005(14) | 0.002(8) | 0.001(5) |
| N9 | 0.069(13) | 0.081(10) | 0.20(2) | 0.028(12) | 0.030(13) | 0.006(8) |
| C9 | 0.044(10) | 0.079(8) | 0.076(17) | 0.027(9) | -0.006(9) | -0.008(7) |
| C8 | 0.059(10) | 0.054(7) | 0.079(18) | 0.030(9) | -0.012(10) | -0.009(7) |
| C7 | 0.031(8) | 0.051(6) | 0.082(11) | 0.028(8) | -0.007(7) | -0.021(6) |
| C6 | 0.037(9) | 0.059(8) | 0.092(15) | 0.013(8) | -0.014(9) | -0.009(7) |
| C1 | 0.040(9) | 0.073(7) | 0.092(16) | 0.021(8) | -0.015(9) | -0.009(6) |
| C10 | 0.034(8) | 0.074(7) | 0.073(16) | 0.027(8) | 0.001(8) | -0.006(6) |
| C5 | 0.081(14) | 0.081(10) | 0.11(2) | 0.037(11) | -0.036(13) | -0.009(9) |
| C4 | 0.052(11) | 0.086(10) | 0.09(2) | 0.036(11) | -0.020(12) | -0.003(8) |
| C3 | 0.086(15) | 0.079(9) | 0.15(3) | 0.029(12) | -0.079(17) | -0.009(9) |
| C2 | 0.053(11) | 0.078(9) | 0.11(2) | 0.014(10) | -0.043(12) | 0.004(8) |
| O10 | 0.019(6) | 0.17(2) | 0.13(2) | -0.047(19) | 0.001(9) | -0.011(9) |
| O11 | 0.072(17) | 0.22(3) | 0.14(3) | 0.01(2) | 0.030(16) | -0.026(18) |
| C11 | 0.021(7) | 0.042(9) | 0.078(10) | 0.024(9) | 0.002(5) | -0.012(6) |
| O12 | 0.08(2) | 0.25(3) | 0.54(11) | 0.07(4) | -0.06(4) | 0.03(2) |
| C12 | 0.013(5) | 0.031(8) | 0.081(10) | 0.031(9) | 0.001(5) | -0.009(6) |
| O13 | 0.30(5) | 0.082(16) | 0.27(6) | 0.00(2) | 0.00(4) | -0.070(18) |
| C13 | 0.017(8) | 0.048(6) | 0.080(10) | 0.015(7) | -0.003(8) | -0.004(5) |
| C14 | 0.010(5) | 0.066(8) | 0.090(19) | 0.037(11) | 0.013(7) | 0.001(5) |
| C15 | 0.007(7) | 0.054(7) | 0.105(12) | 0.025(8) | 0.010(7) | -0.002(5) |
| C16 | 0.012(6) | 0.103(14) | 0.101(12) | 0.023(10) | 0.004(7) | -0.004(8) |
| C17 | 0.032(11) | 0.147(14) | 0.110(15) | 0.020(15) | -0.005(11) | -0.008(10) |
| C18 | 0.08(2) | 0.163(16) | 0.18(3) | 0.05(2) | -0.003(18) | -0.017(14) |
| C19 | 0.061(11) | 0.056(7) | 0.088(17) | 0.001(8) | -0.008(12) | -0.013(6) |
| C20 | 0.064(12) | 0.063(9) | 0.12(2) | 0.004(11) | -0.012(11) | -0.004(7) |
| C21 | 0.048(11) | 0.060(10) | 0.14(2) | 0.017(11) | -0.011(10) | -0.024(7) |
| C22 | 0.042(11) | 0.076(13) | 0.14(2) | 0.018(12) | -0.013(10) | -0.026(8) |
| C23 | 0.038(10) | 0.084(12) | 0.14(2) | 0.026(13) | -0.031(11) | -0.054(7) |
| C24 | 0.051(12) | 0.087(14) | 0.19(3) | 0.051(16) | -0.010(13) | -0.046(8) |
| C25 | 0.056(12) | 0.061(12) | 0.20(2) | 0.051(13) | -0.001(12) | -0.045(8) |
| C26 | 0.071(13) | 0.054(11) | 0.20(2) | 0.041(13) | 0.021(12) | -0.019(8) |
| C28 | 0.003(7) | 0.052(7) | 0.117(13) | 0.023(9) | -0.002(8) | 0.009(6) |
| C29 | 0.025(8) | 0.039(9) | 0.117(13) | 0.014(9) | 0.002(8) | -0.018(5) |
| C30 | 0.056(12) | 0.070(12) | 0.102(17) | 0.011(13) | -0.019(11) | -0.012(8) |
| C31 | 0.061(12) | 0.114(17) | 0.098(18) | 0.003(16) | -0.019(12) | -0.030(9) |
| C32 | 0.072(12) | 0.055(12) | 0.13(2) | 0.006(12) | 0.015(12) | -0.055(8) |
| C33 | 0.062(11) | 0.042(10) | 0.143(19) | 0.007(11) | 0.032(11) | -0.045(7) |
| C34 | 0.012(8) | 0.074(11) | 0.141(18) | 0.015(12) | 0.018(10) | -0.023(6) |
| C35 | 0.038(12) | 0.173(15) | 0.14(2) | 0.019(16) | -0.013(14) | -0.023(10) |
| C38 | 0.13(3) | 0.14(2) | 0.28(5) | 0.04(3) | 0.00(3) | -0.041(16) |
| C39 | 0.08(2) | 0.23(3) | 0.31(6) | 0.12(4) | 0.00(3) | 0.04(2) |
| C40 | 0.13(3) | 0.21(2) | 0.17(5) | 0.06(3) | 0.02(3) | 0.04(2) |
| C36 | 0.033(11) | 0.170(14) | 0.11(3) | 0.006(16) | -0.052(11) | -0.035(10) |
| C37 | 0.069(16) | 0.18(2) | 0.18(4) | 0.05(2) | 0.000(18) | 0.001(16) |
| C42 | 0.11(2) | 0.17(2) | 0.15(4) | 0.04(2) | -0.056(18) | -0.025(15) |
| C41 | 0.076(14) | 0.091(14) | 0.13(2) | 0.017(15) | -0.001(14) | 0.015(10) |
| C49 | 0.096(14) | 0.038(8) | 0.09(2) | 0.016(10) | -0.035(13) | -0.003(8) |
| C50 | 0.085(13) | 0.049(7) | 0.07(2) | 0.017(10) | -0.023(12) | -0.010(7) |
| C51 | 0.030(8) | 0.041(6) | 0.087(12) | 0.007(7) | -0.002(8) | 0.013(5) |
| C52 | 0.032(9) | 0.048(8) | 0.107(16) | 0.026(9) | -0.018(9) | -0.016(7) |
| C43 | 0.037(9) | 0.039(6) | 0.101(17) | 0.016(8) | -0.017(9) | -0.007(6) |
| C48 | 0.052(9) | 0.032(6) | 0.075(16) | 0.010(8) | -0.013(9) | 0.000(5) |
| C47 | 0.055(10) | 0.065(9) | 0.069(17) | 0.014(10) | -0.011(10) | -0.016(8) |
| C46 | 0.041(10) | 0.065(9) | 0.08(2) | 0.018(11) | -0.023(11) | -0.031(7) |
| C45 | 0.086(14) | 0.076(9) | 0.12(3) | 0.043(13) | -0.055(15) | -0.049(9) |
| C44 | 0.15(2) | 0.057(11) | 0.20(3) | 0.058(15) | -0.12(2) | -0.060(12) |
| C53 | 0.027(8) | 0.036(8) | 0.081(11) | 0.007(9) | 0.007(5) | 0.005(6) |
| C54 | 0.009(5) | 0.041(9) | 0.084(11) | 0.013(9) | 0.004(5) | 0.002(6) |
| C55 | 0.001(7) | 0.085(7) | 0.079(11) | 0.013(8) | 0.005(8) | -0.001(6) |
| C56 | 0.001(5) | 0.090(7) | 0.13(2) | -0.005(10) | 0.006(8) | -0.003(5) |
| C57 | 0.009(8) | 0.084(8) | 0.135(14) | 0.009(9) | 0.006(9) | 0.002(6) |
| C58 | 0.019(7) | 0.105(15) | 0.136(14) | 0.006(11) | 0.008(8) | -0.001(9) |
| C59 | 0.002(8) | 0.191(16) | 0.139(16) | 0.024(17) | 0.011(12) | -0.006(11) |
| C60 | 0.034(13) | 0.189(15) | 0.14(3) | 0.031(17) | 0.018(14) | 0.000(13) |
| C61 | 0.046(10) | 0.095(8) | 0.082(17) | 0.014(10) | -0.019(12) | 0.002(7) |
| C62 | 0.044(11) | 0.090(8) | 0.14(2) | 0.008(9) | 0.014(11) | -0.001(7) |
| C73 | 0.056(10) | 0.036(8) | 0.121(19) | 0.030(8) | -0.002(9) | 0.035(7) |
| C74 | 0.068(12) | 0.064(9) | 0.14(2) | 0.005(11) | 0.001(13) | 0.021(8) |
| C75 | 0.060(12) | 0.054(9) | 0.16(2) | 0.009(11) | -0.019(12) | 0.028(7) |
| C76 | 0.061(12) | 0.040(8) | 0.18(2) | 0.029(9) | -0.012(11) | 0.024(8) |
| C64 | 0.056(12) | 0.053(8) | 0.18(2) | 0.023(9) | 0.014(11) | 0.016(7) |
| C63 | 0.047(10) | 0.050(8) | 0.126(18) | 0.030(8) | -0.005(9) | 0.018(6) |
| C65 | 0.031(9) | 0.089(10) | 0.15(2) | 0.027(11) | 0.011(12) | 0.018(8) |
| C66 | 0.033(10) | 0.079(11) | 0.156(17) | 0.031(12) | 0.014(10) | -0.003(8) |
| C70 | 0.059(12) | 0.087(14) | 0.18(2) | 0.055(15) | 0.033(14) | 0.038(8) |
| C71 | 0.045(12) | 0.118(17) | 0.18(2) | 0.060(17) | 0.051(13) | 0.012(9) |
| C72 | 0.062(11) | 0.073(13) | 0.18(2) | 0.059(14) | 0.037(13) | 0.021(9) |
| C67 | 0.035(9) | 0.065(11) | 0.160(16) | 0.046(12) | 0.028(10) | 0.027(6) |
| C68 | 0.056(12) | 0.071(12) | 0.16(2) | 0.025(13) | 0.028(12) | 0.029(8) |
| C69 | 0.073(13) | 0.082(15) | 0.16(2) | 0.034(14) | 0.037(14) | 0.024(9) |
| C77 | 0.026(13) | 0.186(16) | 0.26(4) | 0.05(2) | 0.044(18) | 0.004(13) |
| C78 | 1.3(4) | 0.24(3) | 1.2(2) | 0.11(7) | 1.0(3) | -0.03(8) |
| C81 | 0.25(6) | 0.22(2) | 0.72(10) | 0.21(5) | 0.29(7) | 0.08(4) |
| C85 | 0.06(2) | 0.26(3) | 0.58(8) | 0.15(4) | 0.09(3) | 0.11(2) |
| C82 | 0.08(2) | 0.23(2) | 0.21(5) | -0.03(3) | -0.03(2) | -0.01(2) |
| C79 | 0.08(2) | 0.24(2) | 0.27(5) | -0.04(3) | 0.05(3) | -0.03(2) |
| C80 | 0.002(8) | 0.193(16) | 0.16(2) | 0.033(19) | 0.002(12) | -0.015(11) |

Table S4. Piezoelectric materials and their maximum piezoelectric response.

| Category | Material | Maximum piezoelectric coefficient (pC/N) |
| --- | --- | --- |
| Inorganic materials | AlN | 5.4 |
|  | ZnO | 9.9 |
|  | Ga_3_AsO_7_ | 23.9 |
| Polymers | PVDF | 32 |
|  | PLA | 10 |
| Biological materials | Pro-Phe-Phe | 3.1 |
|  | Collagen | 12 |
|  | Lysozyme | 6.5 |
|  | L-Aspartate | 13 |
|  | L-Cysteine | 11 |
|  | γ-Glycine | 9.9 |

Table S5. Predicted piezoelectric property of Nap-wfy crystals, including maximum piezoelectric stress constants e*_ij_* (C/m^2^), maximum piezoelectric strain constants d*_ij_* (pC/N), maximum piezoelectric voltage constants g*_ij_* (mVm/N), and minimum elastic constants C*_ij_* (GPa).

| Crystal name | \|e*_ij_*\|*_max_* | \|d*_ij_*\|*_max_* | \|g*_ij_*\|*_max_* | \|C*_ij_*\|*_min_* |
| --- | --- | --- | --- | --- |
| **Nap-wfy** | 0.045 | 21.3 | 912 | 0.151 |

Table S6. Comparison of power outputs of piezoelectric nanogenerators based on different materials.

| Type | | Power density (µW/cm^2^) | Reference |
| --- | --- | --- | --- |
| Biomolecule | Chitin | 0.184 | [23] |
|  | D-BIO | 0.061 | [24] |
|  | Diphenylalanine | 0.122 | [25] |
| Hybrid piezoelectric systems | Vanadium doped  ZnO/bacterial | 0.060 | [26] |
|  | MXene/PVDF | 0.0435 | [27] |
|  | [Cu(L-phe) (Bpy)] /PVDF | 0.85 | [28] |
| Nap-wfy | | 0.475 | this work |
